# Next-Generation Sequencing (NGS) Identified Species-Specific SSR and SNP Markers, Allow the Unequivocal Identification of Strawberry Tree (*Arbutus unedo* L.) Germplasm Accessions and Contribute to Assess Their Genetic Relationships

**DOI:** 10.3390/plants12071517

**Published:** 2023-03-31

**Authors:** Ricardo Pereira, Isabela Anjos, João Reis, Carolina Dias, José Leitão

**Affiliations:** 1 MED-Mediterranean Institute for Agriculture, Environment and Development, CHANGE-Global Change and Sustainability Institute, Faculdade de Ciências e Tecnologia, Campus de Gambelas, Universidade do Algarve, 8005-139 Faro, Portugal; ricper1990@gmail.com (R.P.); iveradosanjos@hotmail.com (I.A.); a61179@ualg.pt (J.R.); a74303@ualg.pt (C.D.); 2 Post-Graduation in Biotechnology and Biodiversity-Rede Pró-Centro Oeste, Universidade do Estado de Mato Grosso, Cáceres 78200-000, Brazil

**Keywords:** strawberry tree, *Arbutus unedo* L., plant germplasm collection, genome assembly, SSR markers, SNP markers, SNP-CAPS markers, RAPD markers, ISSR markers, plant molecular fingerprints, plant genetic relationships

## Abstract

The strawberry tree (*Arbutus unedo* L.), an evergreen bush to small tree of the Ericaceae family, is a main component of the natural flora of the Mediterranean basin that also grows profusely through the Iberian Peninsula, southwestern France, and Ireland. The small edible red fruits are usually used to produce preserves, jams, and liquors, as the Portuguese “aguardente de medronho”. The leaves and fruits have been used for a long time in traditional medicine, and their bioactive compounds are presently the subject of intense research. A strawberry tree germplasm collection was recently established by the company Corte Velada (Odiáxere, Portugal). A set of 50 germplasm accessions was selected for a breeding program. A next-generation sequencing project was performed, resulting in the establishment of the first strawberry tree genome assembly and further identification of 500 SSR and 500 SNP loci. Individual molecular fingerprints for the unequivocal identification of the selected 50 accessions were established based on 71 markers alleles amplified by 4 SSR and 9 SNP markers. The same species-specific markers alleles combined with 61 random amplified markers amplified by 5 RAPD and 5 ISSR primers were used to assess the genetic variability and genetic relationships among the selected accessions.

## 1. Introduction

The strawberry tree (*Arbutus unedo* L.), a diploid (2n = 26) evergreen shrub to medium size tree that belongs to the Ericaceae family, is an important constituent of the natural flora that grows around the Mediterranean basin throughout the Atlantic coast of the Iberian Peninsula, southwestern France, and southwestern Ireland [1].

The produced edible, light to dark red small fruits are mostly consumed fresh after harvest or used for the preparation of preserves and jams and distillates as the “Koumaro” in Greece [2] or the “aguardente de medronho” in Portugal.

Long time used for human and well-being purposes, the strawberry tree leaves and fruits are presently the subjects of a profusion of studies on their chemical and biochemical composition and respective biological activities [3,4,5,6,7], which have already extended to roots [8] and derived products (e.g., distillates) [2,9]. All this research activity has led to the publication of several reviews on this wide topic [10,11]. The relationship between the chemical and biochemical content of the leaves and the physiological response of the plants to abiotic stress was recently assessed [12].

In Portugal, where the strawberry tree grows naturally throughout almost all the country, this species has also been cultivated in small orchards of seed-originated trees, mostly in the hill and mountain areas of the southern Algarve region, often in consociation with other Mediterranean species (e.g., *Quercus* sp.).

In recent years, this paradigm started to change with the establishment of large strawberry tree orchards by modern enterprises, orientated to the commercialization of young plants and to liquor production. Nevertheless, the massive fruit production for the fresh market has main constraints to overcome: the fragility, short consumption period, and short shelf life of the strawberry tree fruits [13].

The use of the strawberry tree as an ornamental plant, both in public places and in private yards, is consistently growing. Contrarily to many other fruit tree crops, the strawberry tree has not been the object of intensive breeding programs, and the available cultivars consist of a small group of clones that exhibit interesting ornamental traits, e.g., different colors of the flowers (cv. Atlantic and cv. Rubra, respectively, exhibiting white vs. red flowers) or compact plant habits (e.g., cv. Compacta, and the even slower growing cv. Elfin King) [14].

**Figure 1:**
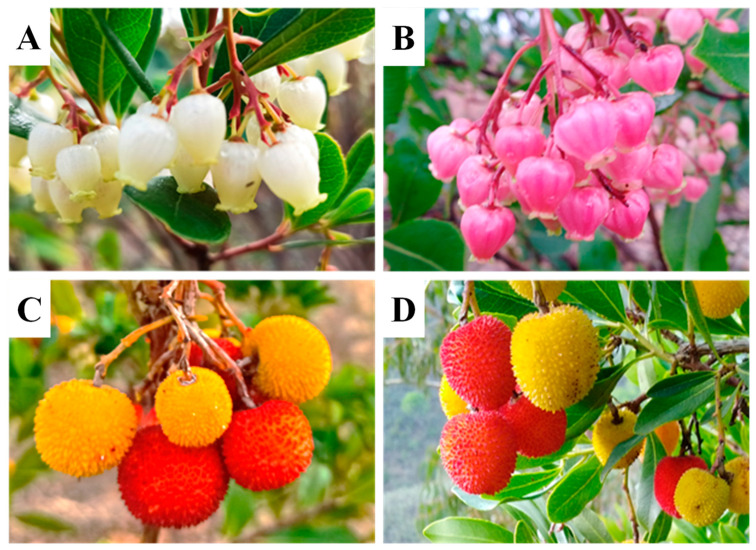
Phenotypic variability within the strawberry tree (*Arbutus unedo* L.) germplasm collection. (**A**,**B**) Flowers with different colors and shapes. (**C**,**D**) Fruits with different forms. Pre-ripened fruits (yellow) and ripened fruits (red).

As the first step in the implementation of a breeding program aimed at the improvement of fruit firmness, and ornamental traits, a strawberry tree field germplasm collection, gathering over 100 accessions (Figure 1), was established in the homestead of the company Corte Velada (Odiáxere, Portugal). From this collection, a set of 50 accessions (Supplementary Table S1) was selected for more accurate observation, analysis, and selection and for genetic improvement via mutation breeding.

Until recently, the genomic data available for strawberry trees (*A. unedo*) were restricted to the chloroplast genome [15], one sequence read archive (SRA) derived from an Ion Torrent random genome sequencing carried out by our laboratory of genomics and genetic improvement (LGGI), Universidade do Algarve, and 1085 short microsatellite sequences retrieved from the same SRA [16]. Presently (accessed on 18 February 2023), the genomic data in the NCBI database consist of 4 additional SRA, 3349 independent sequences, and 1 genome assembly, as well as wide genomic information regarding associated microbiota, etc., reflecting the growing interest in this neglected fruit crop.

Strawberry tree studies using genomic tools, particularly DNA markers techniques, are relatively scarce and generally use randomly amplified markers: RAPD, ISSR, and AFLP, to assess the genetic variability and population structure of the species at the regional level [17,18,19] or covering its whole geographic range [20], or for discrimination from related species [21]. In one of these studies [19], cross-species SSR markers developed for *Vaccinium* spp. were also used to assess *A. unedo* genetic relationships.

The use of species-specific markers is even more limited. SSR markers retrieved from the chloroplast genome have been used to study the spatial distribution of the strawberry tree genetic variation in Portugal [22], while a set of SSR markers retrieved from a first (Ion Torrent) genome sequencing project (www.ncbi.nlm.nih.gov/sra/SRX341237, accessed on 18 February 2023) was validated for wide genomic studies [16].

Herein, we report a second, deeper (Illumina) next-generation sequencing project of the strawberry tree (*Arbutus unedo* L.), the identification of 500 simple sequence repeats (SSR) and 500 single nucleotide polymorphisms (SNP) loci, and the utilization of the 53 markers/alleles amplified by 4 SSR markers and 18 markers/alleles amplified by 9 SNP markers to establish individual and unequivocal molecular fingerprints for a set of 50 germplasm accessions selected for a breeding program. The 71 SRR plus SNP markers/alleles combined with 61 markers amplified by 5 RAPD and 5 ISSR primers were used to assess the genetic diversity and genetic relationships among the selected 50 accessions.

## 2. Results

### 2.1. Establishment of the First Genome Assembly (Scafold) of Arbutus unedo L. by Next-Generation Sequencing (NGS)

Genomic DNA of a selected accession (Golias) extracted from partially purified leaf nuclei of the strawberry tree (*Arbutus unedo* L.) was sent to the company STAB VIDA, Lisboa, Portugal, for (Illumina HiSeq) next-generation sequencing.

Using the Genomics Workbench v.12.0.3, the next-generation sequenced 15.5 Gb of genomic DNA were assembled in 145,873 contigs totaling over 452M nucleotides (N50 = 6250; L50 = 16,158), uploaded to the National Center for Biotechnology Information (NCBI) database (https://www.ncbi.nlm.nih.gov/assembly/GCA_014822125.1 accessed on 18 February 2023) as the first *A. unedo* genome assembly, and named as “UAlgCV_Aunedo_01” according to the involved organizations, the Universidade do Algarve (UAlg) and the company Corte Velada (CV), and the species name (*A. unedo*).

### 2.2. Unequivocal Identification of a Selected Set of 50 Germplasm Accessions of Arbutus unedo L. by Species-Specific SSR and SNP-CAPS Markers

The results of a previous Ion Torrent next-generation sequencing of A. unedo genomic DNA, including the sequences of 1085 microsatellite (SSR) loci (GenBank: KF023636 to KF024720), were uploaded by our lab (LGGI) to the NCBI database in 2013 (www.ncbi.nlm.nih.gov/sra/SRX341237 accessed on 18 February 2023). However, the sequences of these loci are too short (~120 nucleotides) and often unsuitable for primer design and establishment of SSR markers. Nevertheless, this circumstance did not hamper the assessment of the different kinds of SSR motifs and their relative frequency in *A. unedo* in other Ericaceae species and in species of other plant families to be performed [16].

Recently, quick and wide mining of the new genome assembly “UAlgCV_Aunedo_01” allowed the identification of 500 novel and was suitable for utilization dinucleotide SSR loci which were uploaded to the NCBI database (GenBank: MT327200 to MT327699), as sequences of approximately 500 nucleotides per locus were enough to allow multiple alternatives of primer design to transform the SSR loci in SSR markers.

**Figure 2:**
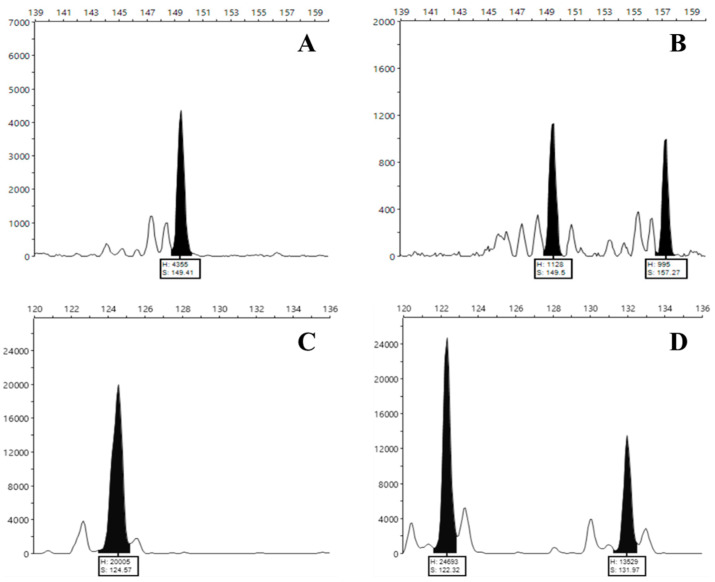
Capillary polyacrylamide gel electropherograms: (**A**,**B**) locus MT327557, (**A**) Homozygous pattern of accession V1; (**B**) Heterozygous pattern of accession AH1; (**C**,**D**) locus MT327513, (**C**) Homozygous pattern of accession AH1; (**D**) Heterozygous pattern of the accession V1. *X* axis: (S)—Fragment size (bp); *Y* axis (H)—Relative Fluorescence Unit (RF).

Primers were designed for 25 novel SSR loci, and after a first round of amplification and analyses by agarose gel electrophoresis, 4 SSR markers that amplified clearer and easier to score products were selected for more accurate analysis using fluorophore-labeled primers (Table 1). The amplification products were assessed by fragment analysis, and the resulting data were analyzed using the Peak Scanner™ Software v. 1.0 (Figure 2, Table 2).

The SSR analysis resulted in the identification of 53 different alleles that allowed the establishment of specific molecular fingerprints (eight numbers) for the unequivocal individual identification of all analyzed accessions (Table 2). The discriminative power of these SSR markers is well evidenced by the dendrogram (Supplementary Figure S1) that displays the established genetic relationships among the selected 50 accessions based on these markers.

The second wide mining of the strawberry tree (*A. unedo*) genome assembly allowed the identification and selection of a set of 500 single nucleotide polymorphisms (SNP) loci, which were also uploaded to the NCBI database as sequences of over 500 nucleotides (GenBank: from OM145479 to OM145978).

Among the retrieved 500 SNP loci, 19 were identified as being restricted differentially by the restriction enzyme TaqI, which could be used for the analysis of these loci as SNP-CAPS markers. After preliminary amplification, TaqI restriction, and analysis by agarose gel electrophoresis, 9 SNP-CAPS markers that produced clearer scorable amplification products and restriction fragments were retained to assess the 50 accessions (Table 3).

**Figure 3:**
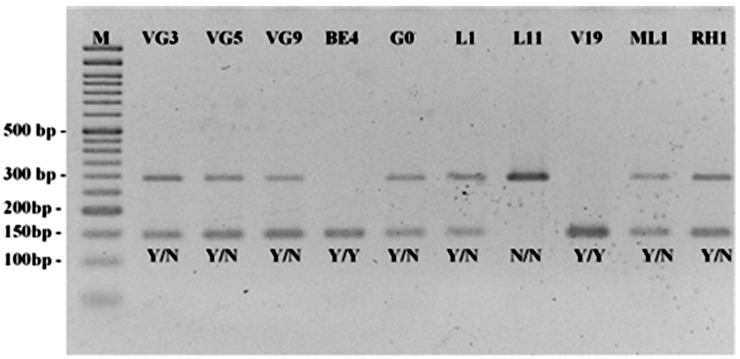
SNP locus OM145551. SNP-CAPS patterns of 10 *A. unedo* accessions using the TaqI restriction enzyme. Y/N—only one allele cut (heterozygous). Y/Y—both alleles cut (homozygous). N/N—Both alleles not cut (homozygous). Notice that the restriction with the enzyme TaqI results in two fragments of, respectively, 145 and 147 nucleotides fused in one single (lower) band. M—Ladder VI (Nzytech, Lisboa, Portugal).

As expected for a diploid species, the genotypes for each SNP locus were: (a) Y/Y, when both alleles were restricted by the restriction enzyme; (b) Y/N, when only one allele was cut, and (c) N/N, when none of the alleles was restricted, and the amplified fragment remained intact (Table 2, Figure 3).

A quick look over the results of the SNP-CAPS analysis (Table 2) reveals a wide panoply of combinations of the identified 18 alleles among the accessions. However, the SNP-CAPS analysis did not result in the molecular discrimination of all accessions since one trio (V17/MA3/VG1) and three pairs of accessions (V13/M9), (V1/M4) and (V7/VG3) exhibit the same molecular patterns (Table 2).

Nevertheless, the combination of the results of the SSR and SNP-CAPS markers allowed the establishment of a relatively easy-to-confirm individual and unequivocal DNA fingerprint, comprising 8 numbers and 18 letters, for the 50 studied accessions (Table 2) that will be applied to all germplasm collection.

### 2.3. Assessment of the Genetic Diversity and Genetic Relationships among 50 Arbutus unedo L. Germplasm Accessions by SSR, SNP-CAPS, RAPD, and ISSR Markers

In combination with random amplified polymorphic DNA (RAPD) and the inter-single sequence repeats markers (ISSR), the SSR and SNP-CAPS markers were also used to assess the genetic diversity and genetic relationships among the selected 50 germplasm accessions.

Estimated based on the identified 53 SSR alleles, the genetic similarity values among the selected 50 accessions ranged from a maximum of 0.75 between two pairs of accessions to very low values as zero or 0.125 (Supplementary Table S2), a circumstance that will be discussed below. The 18 SNP-CAPS alleles also revealed a wide genetic diversity among the same accessions, with genetic similarity values varying from 0.316 to 1.000 (Supplementary Table S3), with the highest value exhibited by the above-mentioned non-discriminated one trio and three pairs of accessions.

The analysis of the combined 71 SSR and SNP-CAPS markers/alleles resulted in genetic similarity values ranging between 0.242 and 0.829 (Supplementary Table S4), which evidence a clear increase of the minimal and lowering of maximal value when the SSR or, respectively, SNP-CAPS markers were used alone, allowing the discrimination of the few accessions not differentiated by the last markers.

**Figure 4:**
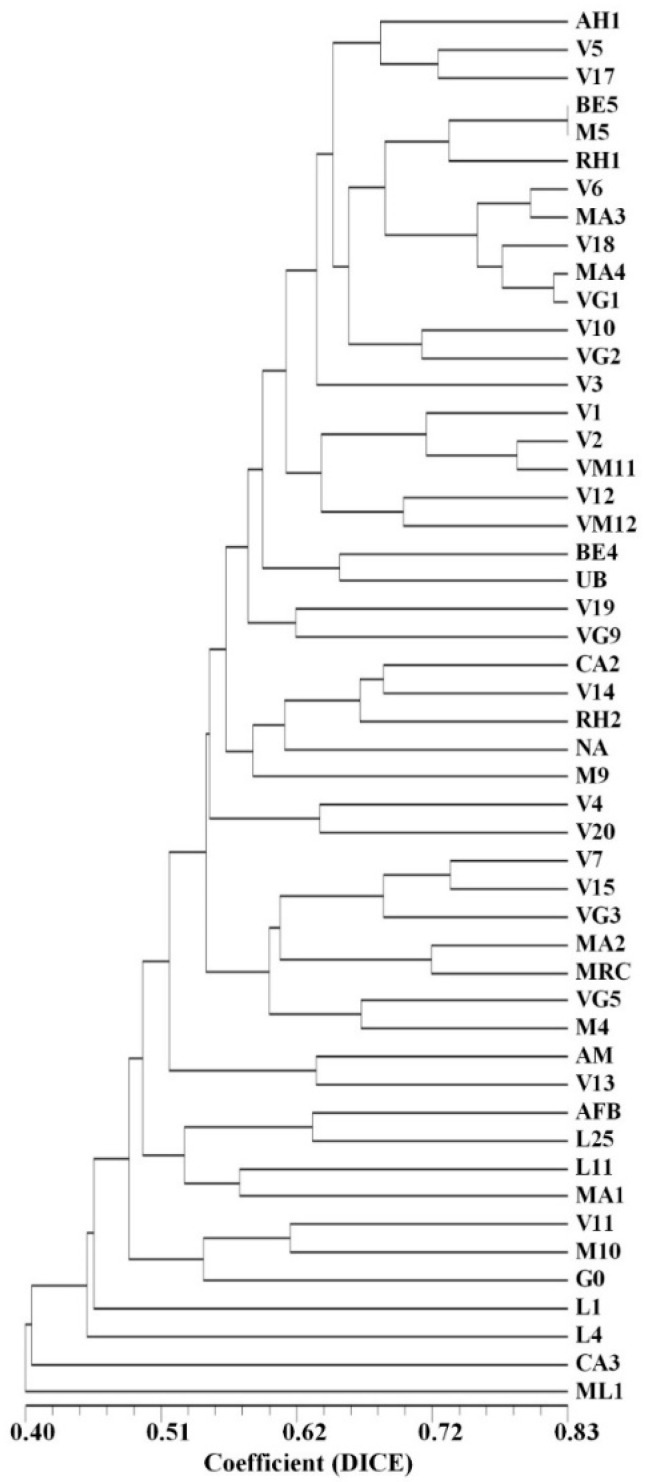
Dendrogram showing the genetic relationships among the selected set of 50 strawberry tree (*Arbutus unedo* L.) germplasm accessions, established based on the merged results of the performed SSR and SNP-CAPS analyses.

The graphic representation of the genetic relationships among the accessions, assessed uniquely by the SSR markers or by the SNP-CAPS markers, is displayed in Supplementary Figure S1. The genetic relationships among the same accessions established using the SSR and SNP-CAPS markers/alleles together are depicted in Figure 4.

Our previous experience indicated that the use of randomly amplified markers to assess the genetic similarity and genetic relationships between individuals of the same species usually results in relatively high absolute genetic similarity values, usually around 0.8 or higher, which are relatively different from the here obtained results with the species-specific SSR and SNP-CAPS markers.

**Figure 5:**
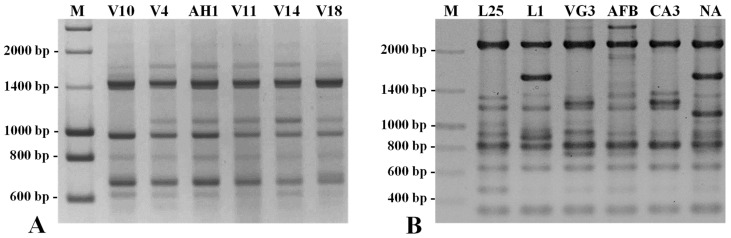
Randomly amplified markers analyzed by agarose gel electrophoresis. (**A**) RAPD patterns of 6 germplasm accessions amplified by primer OPAN11. (**B**) ISSR patterns of 6 germplasm accessions amplified by primer (GA)_8_YC. Notice that the sequence of this primer consists eight repeats of “GA” followed by a pyrimidine (Y) and a cytosine. Notice the absence or the presence of polymorphic bands in specific genotypes (accessions). M—Ladder III (Nzytech, Lisboa, Portugal).

Aiming to solve this discrepancy and improve the calculation of the genetic similarity values and eventual negative effects in the estimation of the genetic relationships among the studied accessions, additional analyses were performed using five random amplified polymorphic DNA (RAPD) and five inter-single sequence repeat (ISSR) primers (Figure 5).

As expected, the genetic similarity values were reckoned based on the clearly amplified and better scorable 27 RAPD and 34 ISSR markers, ranging from 0.795 (accessions L1/AFB) to 0.970 (accessions V1/ V11 and V6/V11) (Supplementary Table S5), which are clearly higher and more acceptable for genotypes of the same species than those calculated based on the SSR and SNP-CAPS markers. Nevertheless, different levels of absolute genetic similarity values do not necessarily imply an alteration of their relative level, which is the base for genetic relationship estimation. A dendrogram depicting the genetic relationships among the 50 accessions estimated based on the results of the RAPD and ISSR analysis can be consulted in Supplementary Figure S1.

To obtain a more consistent evaluation of the genetic similarity and genetic relationships among the studied accessions, a new calculation was performed joining all the 132 (SSR, SNP, RAPD, and ISSR) markers and the resulting genetic similarity values (Supplementary Table S6) that varied from 0.646 (accessions ML1/V3) to 0.884 (accessions MA4/V18), although lower than the obtained based uniquely on randomly amplified markers, are still perceived as acceptable for individuals of the same species.

**Figure 6:**
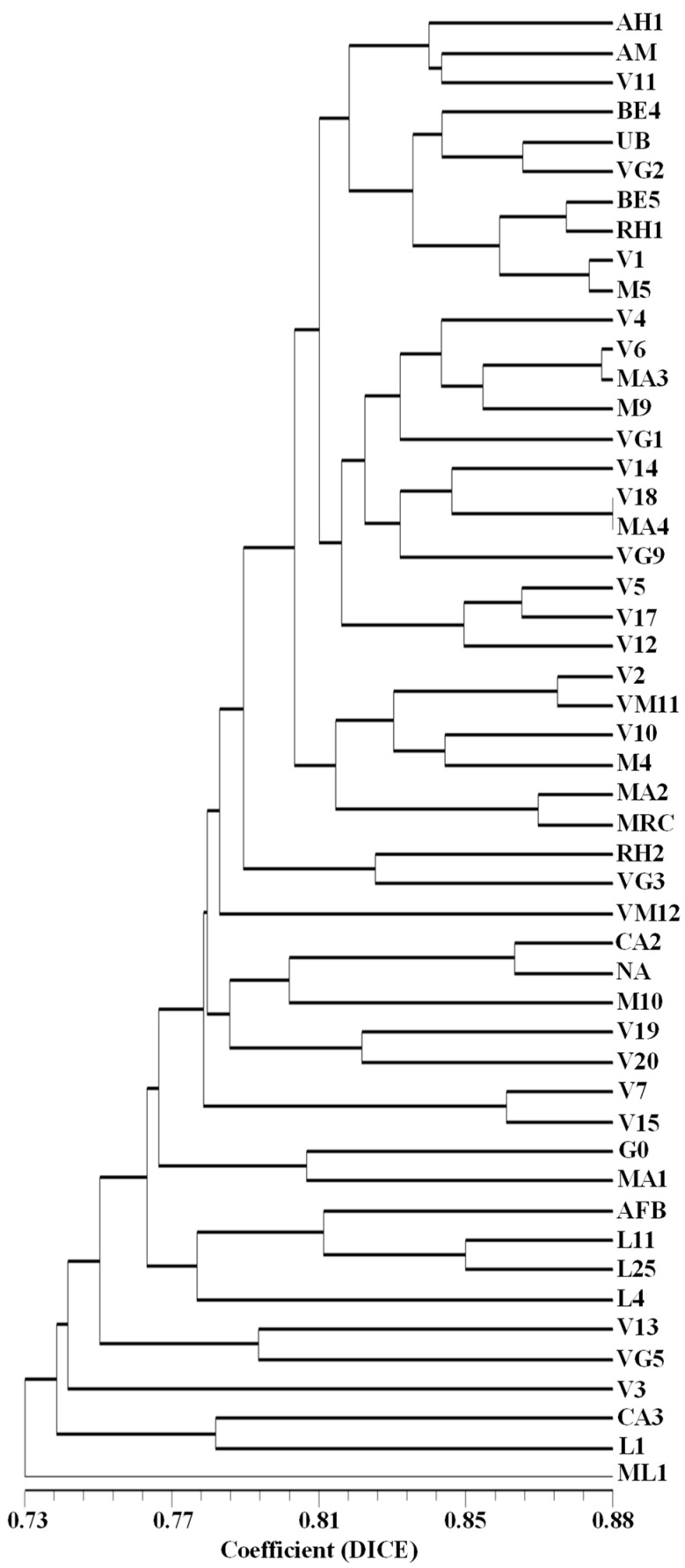
Dendrogram depicting the genetic relationships among the set of 50 strawberry tree *(Arbutus unedo* L.) germplasm accessions selected for a breeding program, established based on the results of species-specific SSR and SNP-CAPS markers and the randomly amplified RAPD and ISSR markers. Notice the calculated higher genetic similarity values compared to the established uniquely based on the SSR and SNP-CAPS markers (Figure 4).

An approximate graphical representation of the genetic relationships among the analyzed 50 accessions is shown in Figure 6. The results of the assessment of the genetic relationships among the studied germplasm accessions need to be taken into consideration in the further accurate selection among the preliminarily chosen 50 accessions. Particular attention needs to be given to those accessions that have demonstrated in some or all analyses high genetic differentiation from the main cluster of accessions, such as ML1, L1, L4, or CA3, though no clear, unique morphological differences have been detected so far in these accessions.

## 3. Discussion

The growing interest in the strawberry tree (*Arbutus unedo* L.) is having a clear impact on the amount of available genomic data and genomic tools, which are continuously increasing. Nevertheless, the above-described genome assembly, as well as the 1585 microsatellites (SSR) loci and the 500 SNP loci uploaded by our lab (LGGI) to the NCBI, is among the main set of molecular data and tools available to the scientific community for genomic studies on this neglected fruit tree species.

So far, the strawberry tree germplasm collection established in the homestead of the company Corte Velada is the only large collection for this species in Portugal, while no information is available on other large collections.

The preliminary phenotypic assessment of this collection allowed the selection of 50 promising accessions for inclusion in a plant breeding program aimed at the identification, or induction via mutation breeding, of clones producing improved fruits for the fresh market or exhibiting novel phenotypes for ornamental purposes.

The SSR (microsatellite) and SNP-CAPS markers analyses allowed the establishment of individual molecular patterns, consisting of 8 numbers and 18 letters, for all analyzed germplasm accessions that allow their unequivocal identification in any stage of the plant material: propagation scions, cuttings, recently rooted or adult plants, or in vitro cultured cells, tissues, or plants.

The SSR markers showed to be more efficient than the SNP-CAPS for that purpose, as total discrimination was achieved by four SSR (PCR) markers, while nine SNP-CAPS markers were not able to discriminate all accessions (Table 2, Supplementary Figure S1). Nevertheless, the joint use of these markers was shown to warrant a high level of discrimination of the plant material and was used to establish an individual molecular fingerprint for the 50 accessions analyzed in this study.

The use of the same SSR and SNP-CAPS markers to assess the genetic similarity relationships among the studied accessions (Figure 3) revealed genetic similarity levels low as zero for SSR markers and 0.316 for SNP-CAPS (Supplementary Tables S4 and S6). It is obviously absurd for two genotypes of the same species to exhibit zero genetic similarity. This result is a consequence of the hyper-polymorphism of the SSR markers, a feature that makes these markers highly efficient for discrimination or for identification of genetic relatedness between individuals, which explains their wide use in forensic issues [23] but makes them not the most adequate for quantification of genetic similarity. These remarks also apply to the SNP-CAPs markers, which, although useful for the identification and determination of the genetic relationships among individuals, are also not the most suitable for their quantification. In fact, although being very accurate for the establishment of genetic relationships, the utilization of SSR or SNP markers for the estimation of genetic similarity estimates, frequently results in very low values (below 0.40) for genotypes of the same plant species, as found for the olive tree [24], *Carica papaya* clones [25], lettuce (*Lactuca sativa* L.) cultivars [26] or *Chrysanthemum* [27].

The use of randomly amplified markers, eventually reinforced by some sequence-specific markers, would be the most suitable approach for more precise quantification of genetic relatedness. In our lab, the use of DNA markers such as random amplified polymorphic DNA (RAPD), inter-single sequence repeat markers (ISSR), and amplified fragment-length polymorphism (AFLP) in multiple plant species, e.g., *Diplotaxis tenuifolia* [28], *Thymus sp.* [29], *Cucurbita pepo* [30], *Phaseolus vulgaris* [31], *Malus domestica* [32], *Ficus carica* [33], etc., resulted consistently in genetic similarity values between specimens of the same species close to, or over 0.8, a value that dropped drastically for individuals of different species. In a study of the genetic diversity among *A. unedo* populations, using RAPD markers and SSR markers developed for *Vaccinium* spp., the enormous discrepancy between the genetic distance values obtained based on the two different types of markers was clearly demonstrated [19].

Nevertheless, it should be stressed that the use of genome-specific molecular markers (e.g., SSR, SNP, etc.), although not the best option for the determination of the genetic similarity values, is a very efficient method for the identification of unique and unequivocal molecular fingerprints of individual genotypes, and very useful for the determination of their relative genetic relationships.

During the last years, novel approaches have been developed for the identification of genetic diversity and genetic relatedness among a large number of genotypes, in particular resorting to novel developments and increasing affordability of the NGS techniques, such as the genotyping by sequencing (GBS) approach.

Nevertheless, the developed in this study, a quick, highly reproducible, and affordable protocol for the unequivocal identification of strawberry tree (*Arbutus unedo* L.) plants, based on SSR and SNP-CAPS markers, can be easily and efficiently used in laboratories of plant production companies and germplasm collection management institutions, for quality control of the produced plants and identification of individual accessions. This protocol is also available for utilization and further development by the strawberry tree (*Abutus unedo* L.) research community.

## 4. Materials and Methods

### 4.1. Plant Material

Fifty accessions of a strawberry tree (*Arbutus unedo* L.) germplasm collection established at the enterprise Corte Velada, Odiáxere, Portugal, were selected (Supplementary Table S1) for a genetic improvement program aimed at the registration as new cultivars producing improved fruits or harboring novel ornamental traits.

Leaf DNA was used for the individual molecular identification of the 50 accessions by SSR and SNP-CAPS markers and for an insight into the genetic diversity and genetic relatedness within this set of selected accessions by these species-specific markers combined with randomly amplified RAPD and ISSR markers.

### 4.2. DNA Extraction

The use of the most common protocols for extraction of high-quality total genomic DNA from *A. unedo* leaves is prevented by the very fast DNA degradation by a still not identified specific DNase activity, which remains strongly active in the presence of EDTA, ionic detergents, as SDS or CTAB, and high temperature. For that reason, we have previously developed a protocol for DNA extraction from relatively purified leaf cell nuclei [16].

For plant molecular characterization, approximately 1 g of leaf material with removed main nervure was ground under liquid nitrogen in a mortar with a pestle. The obtained fine powder was transferred to glass centrifuge tubes containing 6 mL of nuclei isolation buffer (50 mM Tris-HCl (pH 8.0), 0.1 M gradient grade sucrose, and 2% Triton X-100) to the final volume of approximately 7 mL. After centrifugation at 80 g for 2 min, the supernatant was transferred to a new tube, and after centrifugation for 5 min at 900 g, the supernatant was discarded, and the enriched with nuclei pellet was used for DNA extraction following protocol 1 of the kit NZY Plant/Fungi gDNA isolation (NZYTech) which uses CTAB as the ionic detergent. The DNA was eluted from the chromatographic microcolumns with 100 µL autoclaved milli-Q water.

### 4.3. DNA Extraction for Next-Generation Sequencing (NGS)

The first steps in the extraction of genomic DNA for Next-Generation Sequencing were as described above. However, after the second centrifugation, 2 µL of the nuclei-enriched pellet was transferred to a glass microscope slide and mixed with 10 µL of a DAPI solution for quality analysis of the nuclei under UV microscopy (Olympus Vanox AHBT3). The bulk of the pellet was resuspended in 2 mL microfuge tubes containing 750 μL of previously heated to 75 °C DNA isolation buffer (300 mM Tris HCl pH 8.0, 25 mM EDTA pH 8.0, 2 M NaCl, 1 mM DTT, 2% CTAB, and 2% PVP) complemented with 250 μg/mL proteinase-K (Sigma-Aldrich, Burlington, MA, USA) and 20 μg/mL RNase A (Sigma-Aldrich). After 10 min incubation at 75 °C, the DNA was extracted twice with chloroform: isoamyl alcohol (24:1), precipitated with 3 volumes of absolute ethanol, and stored in 75% absolute ethanol at −20 °C. For use in subsequent procedures, the precipitated DNA was centrifuged for 5 min at 13,000 rpm, the supernatant was discarded, and the pellet was washed with 500 µL 75% absolute ethanol. After the new centrifugation, the supernatant was discarded, and the pellet was left to dry in the centrifuge tubes for 2 h. The DNA was slowly resuspended in 50 µL autoclaved milli-Q water for some days in a refrigerator.

### 4.4. Quality Evaluation and Quantification of the Extracted DNA

The integrity, eventual contamination with RNA, and the first approximate quantification of the extracted DNA were assessed by agarose gel (1.4%) electrophoresis. The DNA concentration was approximately determined in the same gels by comparison with different known amounts of genomic DNA extracted from *Pisum sativum* roots, which do not contain chlorophyll or other pigments that can bias the spectrophotometry results. A more accurate quantification was then obtained by UV spectrophotometry (NanoDrop One, Thermofisher), whose results were accepted if falling within the concentration limits established by comparison with the *Pisum* samples in agarose gels. The amplifiability of the DNA samples was assessed by RAPD-PCR using a cocktail of three primers.

### 4.5. NGS Sequencing

After slow resuspension in autoclaved milli-Q water, the DNA integrity and purity were assessed by agarose gel electrophoresis, as described above. After quantification by UV spectrophotometry (NanoDrop One), a DNA sample (50 µL, 65.80 ng/µL) was sent to the company STAB VIDA, Lisboa, Portugal, for next-generation (Illumina HiSeq platform) sequencing using 150 bp paired-end sequencing reads. After a new quality analysis of the DNA samples by agarose (1.5%) electrophoresis and quantification using a Qubit 2.0 Fluorometer (Thermo Fisher Scientific, Waltham, MA, USA) with a Qubit dsDNA BR Assay kit, the library was generated using the Kapa HyperPrep kit (Roche, Basel, Switzerland). The analysis of the generated sequence raw data and the de novo assembly were carried out using the software CLC Genomics Workbench v.12.0.3 [34], QUAST 5.0.2 [35], BUSCO [36], and an algorithm based on de Bruijn graphs [37].

The confirmation of the integrity and purity of the DNA sample was performed by 1.5% agarose gel electrophoresis and the quantity by Qubit analysis. The quality of the produced data was determined by the Phred quality score at each cycle. The plot containing the average quality at each cycle was created with FastQC [38]. The trimming of the raw sequences was performed using the parameters: (i) ambiguous limit (2 nucleotides); (ii) quality limit 0.01; (iii) Minimum number of nucleotides in reads (50 nucleotides). (iv) Discard short reads (yes). The trimmed sequence reads were used to perform a de novo assembly using an algorithm based on de Bruijn graphs [37]. After the initial contig creation, the reads were mapped back to the contigs for assembly correction using the following parameters (and values): (i) length fraction = 0.8; (ii) Similarity fraction = 0.8; (iii) Minimum contig size = 500 bp; (iv) Minimum coverage = 5x. The software QUAST 5.0.2 [35] and BUSCO [36] were used to perform a quality assessment and evaluation of the genome assembly.

When needed (e.g., confirmation of SSR primers and markers sequences), the detailed analysis of the sequence contigs and respective reads was performed using the software Tablet 1.21.02.08 [39].

### 4.6. Primer Design and Synthesis

The FastPCR 6.7 Software (PrimerDigital, Helsinki, Finland) [40] was used for primers design and calculation of their parameters and eventual self- or pair-annealing according to the lab rules for primers design: 16–20 nucleotides long, ~50% Gs and Cs and melting temperature ~50 °C. Common, non-labeled primers were synthesized by the company Eurofins Genomics (Ebersberg, Germany). The fluorescent-labeled primers were ordered from the company STAB VIDA, Lisboa, Portugal.

### 4.7. Single-Sequence Repeats (SSR) Markers Analysis

Five hundred dinucleotide SSR loci (~500 bp sequences containing an SSR motif) were identified and selected by a manual random search for microsatellite motifs (at least six repeats of the dinucleotide) within multiple genome contigs. The primers for the amplification of the respective SSR markers were designed for the amplification of 100–200 nucleotide-long products.

The amplification of the SSR (microsatellite) markers was performed in 30 µL reactions, starting with an initial denaturation at 94 °C for 1 min and 30 s, followed by 35 cycles of 30 s denaturation at 94 °C, 30 s annealing at a temperature that varied according to the specific primer pair, and 1 min extension at 72 °C, followed by a final extension period of 10 min at 72 °C.

The PCR products were analyzed by 3% agarose gel electrophoresis, and the markers that produced more clearly amplified bands were chosen for amplification with fluorochrome-labeled primers. Half (15 µL) of the amount of the amplifications performed using a labeled forward primer was first analyzed by agarose gel electrophoresis, and the second half of the approved samples were sent to the company STAB VIDA for fragment analysis by capillary polyacrylamide gel electrophoresis.

The analysis of the amplified fragments was performed in a 3730XL Genetic Analyzer platform using GeneScan™ 500 LIZ™ as the dye size standard, and the resulting data were analyzed using the Peak Scanner™ Software v. 1.0 (Applied Biosystems, Thermo Fisher, Waltham, MA, USA).

### 4.8. Single Nucleotide Polymorphisms (SNP) Markers Analysis

Five hundred SNP loci (~500 bp sequences containing an SNP) were identified among the genome assembly contigs using the Geneious Prime v.2021.2.5 software (Biomatters, Auckland, New Zealand).

The seven-nucleotide sequence that contained the identified SNP in the 4th nucleotide was analyzed by the NEBcutter V2.0 software (New England Biolab, Ipswich, MA, USA) [41] for the identification of restriction enzymes that differentially cut the alternative SNP alleles.

Nineteen SNP markers, identified as harboring the SNP within a sequence recognized by the restriction enzyme TaqI, were selected for further analysis. The primers for analysis of the SNP markers were designed for amplification of products between 200–300 nucleotides which, when assessed as cleaved amplified polymorphic sequences (CAPS) markers (SNP-CAPS) using the enzyme TaqI, originate clearly visible restriction fragments. The PCR protocol was the same used for SSR markers. Fifteen microliters of the amplified products were analyzed by 3% agarose gel electrophoresis. The remaining 15 µL of well-amplified samples were cut using the TaqI restriction enzyme, and the restriction products were analyzed by 3% agarose gel electrophoresis.

The species-specific condition of the used SSR and SNP-CAPS markers was reconfirmed recently by the non-identification of any significantly similar sequences than among the uploaded by our laboratory sequencing data (https://blast.ncbi.nlm.nih.gov/Blast.cgi, accessed on 31 January 2023).

### 4.9. Random Amplified Polymorphic DNA (RAPD) and Inter-Single Sequence Repeated (ISSR) Markers Analyses

The random amplified polymorphic DNA (RAPD) analyses were performed using the primers OPAL07, OPAL12, OPAM10, OPAM14, and OPAN11 (Operon Technologies, Alameda, CA, USA) and the inter-single sequence repeats (ISSR) analyses using the primers (GA)_8_YT, (GA)_8_YC, (GA)_8_YG, (AG)_8_YT and (AG)_8_YC (ordered from Nzytech, Lisboa, Portugal). The procedures for RAPD and ISSR analyses were previously described in [42].

### 4.10. Additional Data Analysis

The NTSYS-pc program [43] was used for cluster analysis. The genetic similarity between the accessions was reckoned using the coefficient DICE [44] by pairwise comparisons based on the percentage of common fragments, according to the equation: similarity = 2 Nab/(Na + Nb), where Nab is the number of scored amplification products simultaneously present in accessions ‘a’ and ‘b’, Na is the number of amplification products scored in accession ‘a’, and Nb is the number of scored fragments in accession ‘b’. The unweighted pair-group method with arithmetic averages (UPGMA) was used to calculate the cophenetic matrix used for dendrogram construction.

## 5. Conclusions

The herein reported research resulted in:

(a) Three major contributions for further genomic studies by the strawberry tree (*Arbutus unedo* L.) research community: (i) the first genome assembly (scaffold) for this fruit tree species; (ii) a set of 500 additional SSR loci; and (iii) a set of 500 SNP loci.
(b) The unequivocal molecular (SSR and SNP-CAPS markers) identification of a set of 50 (*A. unedo*) germplasm accessions selected for a plant breeding program.
(c) The assessment of the genetic variability and genetic relationships among the same selected set of germplasm accessions using SSR, SNP-CAPS, RAPD, and ISSR markers.
(d) The development of a fast, easy to perform and affordable protocol, based on SSR and SNP-CAPS markers, that will be used for identification of all accessions registered in the Corte Velada germplasm collection and is available for plant identification or other purposes by the strawberry tree research community.

## Figures and Tables

**Table 1:** SSR markers used for molecular identification of *A. unedo* clones.

Loci (GenBank)	Primers	5′ Fluorophore Modification
MT327202	Fw_CACCGCAACTTCCTAA *	Atto 550
	Rv_CTCAACTTTCTAAACGTCAC	
MT327224	Fw_ACCACTCTTTGTCTCC *	Hex
	Rv_TTGGCAAATGTATTACGG	
MT327513	Fw_TCTAGTTCGAGACTCTAAGC *	6-FAM
	Rv_ACGAATCGAATCAGATTGAC	
MT327557	Fw_AACTTAGATTTGGCATGAAG *	Atto 565
	Rv_ACATTGGACTGTTTAGATCA	

* Labeled primer.

**Table 2:** Molecular fingerprints of the 50 accessions.

Accessions	MT327224	MT327202	MT327557	MT327513	OM145552	OM145840	OM145884	OM145971	OM145551	OM145712	OM145708	OM145595	OM145977
**AH1**	166;174	170;182	149;157	124;124	Y/Y	Y/Y	Y/Y	Y/Y	N/N	Y/Y	Y/Y	N/N	Y/Y
**AFB**	166;180	162;162	147;147	122;124	Y/Y	Y/N	N/N	Y/Y	N/N	Y/Y	N/N	Y/N	Y/Y
**AM**	164;174	164;174	137;157	120;120	Y/N	Y/Y	Y/N	Y/Y	N/N	Y/Y	N/N	Y/N	Y/Y
**BE4**	172;174	164;168	149;149	126;132	Y/N	Y/Y	Y/Y	Y/Y	Y/Y	Y/Y	N/N	Y/Y	N/N
**BE5**	170;174	172;176	149;149	124;124	Y/Y	Y/N	Y/Y	Y/Y	N/N	Y/Y	N/N	Y/Y	Y/Y
**CA2**	164;170	166;180	159;159	120;120	Y/Y	Y/N	N/N	Y/Y	N/N	Y/Y	N/N	Y/N	Y/N
**CA3**	176;176	168;172	139;159	122;130	N/N	Y/Y	Y/Y	Y/Y	Y/N	N/N	N/N	N/N	Y/N
**G0**	164;172	164;180	159;159	132;132	Y/N	Y/N	Y/N	Y/N	Y/N	Y/N	Y/N	Y/N	Y/N
**L1**	168;170	168;178	159;163	134;134	N/N	Y/Y	Y/N	N/N	Y/N	Y/Y	N/N	Y/N	Y/N
**L4**	164;166	170;170	137;149	122;124	N/N	Y/Y	N/N	N/N	Y/N	Y/Y	N/N	Y/Y	Y/N
**L11**	160;168	172;180	159;159	124;124	Y/Y	Y/N	Y/N	N/N	N/N	Y/Y	N/N	Y/N	Y/N
**L25**	164;166	176;192	147;147	120;124	Y/Y	Y/Y	Y/N	N/N	N/N	Y/Y	N/N	Y/N	Y/N
**M4**	160;168	174;178	157;157	122;138	Y/Y	Y/Y	N/N	Y/Y	Y/N	Y/Y	N/N	Y/Y	Y/N
**M5**	170;174	172;178	149;159	124;124	Y/Y	Y/N	Y/N	Y/Y	Y/N	Y/Y	N/N	Y/Y	Y/N
**M9**	166;170	168;168	159;159	132;132	Y/N	Y/Y	Y/Y	Y/Y	N/N	Y/Y	N/N	N/N	Y/Y
**M10**	166;166	176;188	137;159	122;132	Y/N	Y/N	Y/N	Y/Y	N/N	Y/Y	Y/N	Y/N	Y/N
**MA1**	168;184	164;168	147;159	124;132	Y/Y	Y/Y	N/N	N/N	N/N	Y/Y	Y/N	Y/Y	Y/Y
**MA2**	168;170	172;182	147;157	124;134	Y/Y	Y/Y	N/N	N/N	Y/N	Y/Y	N/N	Y/N	Y/N
**MA3**	162;164	166;172	137;159	122;132	Y/Y	Y/Y	Y/Y	Y/Y	N/N	Y/Y	Y/N	Y/N	Y/Y
**MA4**	174;184	172;180	149;159	122;136	Y/Y	Y/Y	Y/Y	Y/Y	N/N	Y/Y	N/N	Y/Y	Y/Y
**MRC**	168;170	164;172	151;157	122;134	Y/Y	Y/Y	N/N	Y/Y	N/N	Y/Y	N/N	Y/N	Y/Y
**ML1**	168;182	154;162	147;157	122;131	N/N	Y/N	N/N	Y/Y	Y/N	Y/N	N/N	Y/N	Y/N
**NA**	168;170	154;166	149;159	121;137	Y/Y	Y/N	Y/N	Y/Y	N/N	Y/Y	N/N	N/N	Y/N
**RH1**	160;174	172;172	141;149	124;132	Y/Y	Y/Y	Y/N	Y/Y	Y/N	Y/Y	Y/N	Y/Y	Y/Y
**RH2**	170;174	168;178	137;159	120;132	Y/Y	Y/Y	N/N	Y/Y	N/N	Y/Y	Y/N	Y/Y	Y/N
**UB**	164;170	164;178	149;149	122;122	N/N	Y/Y	Y/Y	Y/Y	N/N	Y/Y	N/N	Y/Y	Y/N
**V1**	170;174	178;188	149;149	122;132	Y/Y	Y/Y	N/N	Y/Y	Y/N	Y/Y	N/N	Y/Y	Y/N
**V2**	168;170	166;178	149;157	132;132	Y/Y	Y/Y	N/N	Y/N	Y/N	Y/Y	N/N	Y/Y	Y/N
**V3**	168;172	172;176	139;139	124;132	Y/Y	Y/Y	Y/Y	Y/N	Y/N	Y/Y	N/N	Y/Y	Y/N
**V4**	166;170	172;172	145;159	122;132	Y/Y	N/N	Y/Y	Y/Y	Y/Y	Y/Y	N/N	Y/Y	N/N
**V5**	164;166	168;180	149;153	124;132	Y/Y	Y/Y	Y/Y	Y/Y	Y/N	Y/Y	Y/N	Y/N	Y/N
**V6**	164;170	176;180	149;159	122;132	Y/Y	Y/Y	Y/Y	Y/Y	N/N	Y/Y	Y/N	Y/Y	Y/Y
**V7**	168;176	164;184	149;159	134;136	Y/Y	Y/Y	N/N	Y/Y	Y/N	Y/Y	N/N	Y/N	Y/N
**V10**	170;176	172;174	137;177	124;136	Y/Y	Y/Y	Y/Y	Y/Y	Y/N	Y/Y	N/N	Y/N	Y/N
**V11**	166;174	164;168	145;145	122;130	Y/N	Y/N	Y/N	Y/Y	Y/N	Y/Y	Y/N	N/N	Y/N
**V12**	172;184	176;182	149;157	132;136	Y/Y	Y/Y	Y/N	Y/Y	N/N	Y/Y	N/N	Y/N	Y/Y
**V13**	164;168	164;164	151;151	121;121	Y/N	Y/Y	Y/Y	Y/Y	N/N	Y/Y	N/N	N/N	Y/Y
**V14**	168;170	172;178	143;159	120;120	Y/Y	Y/Y	Y/Y	Y/Y	N/N	Y/Y	Y/N	Y/N	Y/N
**V15**	166;168	172;172	149;159	134;134	Y/Y	Y/Y	N/N	Y/Y	Y/Y	Y/Y	N/N	Y/N	N/N
**V17**	172;174	164;180	137;161	124;132	Y/Y	Y/Y	Y/Y	Y/Y	N/N	Y/Y	Y/N	Y/N	Y/Y
**V18**	168;184	172;172	149;149	122;132	Y/Y	Y/Y	Y/Y	Y/Y	Y/N	Y/Y	N/N	Y/Y	Y/Y
**V19**	164;168	172;178	149;157	120;122	Y/N	Y/Y	Y/N	Y/Y	Y/Y	Y/Y	N/N	Y/N	N/N
**V20**	160;168	168;172	137;159	130;134	Y/Y	Y/N	Y/N	Y/Y	Y/Y	Y/Y	Y/N	Y/Y	N/N
**VG1**	170;184	172;182	149;159	122;124	Y/Y	Y/Y	Y/Y	Y/Y	N/N	Y/Y	Y/N	Y/N	Y/Y
**VG2**	168;170	168;178	137;149	122;136	Y/Y	Y/N	Y/Y	Y/Y	Y/N	Y/Y	N/N	Y/Y	Y/N
**VG3**	172;184	164;178	149;159	120;120	Y/Y	Y/Y	N/N	Y/Y	Y/N	Y/Y	N/N	Y/N	Y/N
**VG5**	160;168	164;184	149;157	124;138	Y/Y	Y/Y	N/N	Y/Y	Y/N	N/N	N/N	N/N	Y/N
**VG9**	166;184	172;178	149;149	120;136	Y/Y	Y/Y	Y/Y	Y/Y	Y/N	N/N	Y/N	Y/N	Y/N
**VM11**	160;170	166;166	149;157	132;132	Y/N	Y/Y	N/N	Y/Y	Y/N	Y/Y	Y/N	Y/N	Y/N
**VM12**	174;184	178;182	149;157	132;132	Y/Y	Y/Y	Y/Y	N/N	Y/N	Y/Y	N/N	Y/Y	Y/N

**Y**—TaqI restricted allele; **N**—TaqI non-restricted allele.

**Table 3:** Analyzed SNP-CAPS markers.

Loci (GenBank)	Primers	T (°C) *	Product Length (bp) (Restriction Fragments)
OM145551	FW: AGAAAGAGCTGAACACG	57	292 (147/145)
	RV: AGTTATTTCCTAGCCGAATC		
OM145552	FW: AAATATCACCACATCGGG	60	218 (135/83)
	RV: GATCAACCCTTTTGTACAC		
OM145595	FW: GTTGGATTTGTGTAGATCATG	57	278 (139/139)
	RV: TTGGTCTCTGGAGTTCTA		
OM145708	FW: GAAGATGATTCAGCATGTTAG	58	258 (182/76)
	RV: TGAAATAAGCAACGGTACA		
OM145712	FW: CAGATATTTGTCCTAACATGAAG	59	273 (143/130)
	RV: GATATGAATAGAACAACGCG		
OM145840	FW: TTCCAGTATAAGTTCTTGGTG	61	291 (154/137)
	RV: CAGGAACCATAAGAATAGTGA		
OM145884	FW: TCTATTGCTGCCAAGTAC	59	272 (154/118)
	RV: TCAAAGGTATAACTGAGGC		
OM145971	FW: ATACTAGGAACTTGGAAGTG	60	296 (155/141)
	RV: AAGTCAAATGGAGTTATTTCC		
OM145977	FW: TTGTAGGAGTACATGGTCT	59	286 (145/141)
	RV: TATTGTGAGCATGGTGATAG		

* Amplification annealing temperature.

## Data Availability

The data on genome assembly, SRA data set; SSR and SNP sequences, were uploaded to the NCBI database and can be accessed using the names or codes provided in the text. Additional data supporting reported results can be found as supplementary tables.

## Supplementary Materials

The following supporting information can be downloaded at: https://www.mdpi.com/article/10.3390/plants12071517/s1, Figure S1: (A) Genetic intraspecific relationships among 50 selected strawberry tree (*Arbutus unedo* L.) germplasm accessions, assessed by SSR markers; (B) Genetic relationships among the same accessions assessed by SNP-CAPS marker; (C) Genetic relationships among the same accessions, assessed by RAPD and ISSR markers. Notice the very low genetic similarity values estimated by SSR markers vs. the estimated by the randomly amplified markers genetic similarity values which, according to multiple studies (see text), are expected to be close or above 0,8 (DICE coefficient); Table S1: Morphologic traits of 50 accessions selected for further breeding; Table S2: Similarity matrix (SSR markers); Table S3: Similarity matrix (SNP-CAPS); Table S4: Similarity matrix (SSR and SNP-CAPS); Table S5: RAPD and ISSR markers; Table S6: SSR, SNP-CAPS, RAPD and ISSR markers.
