# Supplementary material for: Next-Generation Sequencing (NGS) Identified Species-Specific SSR and SNP Markers, Allow the Unequivocal Identification of Strawberry Tree (Arbutus unedo L.) Germplasm Accessions and Contribute to Assess Their Genetic Relationships
Source: Plants (Basel). 2023 Mar 31;12(7):1517. doi: 10.3390/plants12071517 (PMC10096993; doi:10.3390/plants12071517)
Supplement: Supplementary file 1 [file plants-12-01517-s001.zip › plants-2275838-Supplementary Figure S1.pdf]

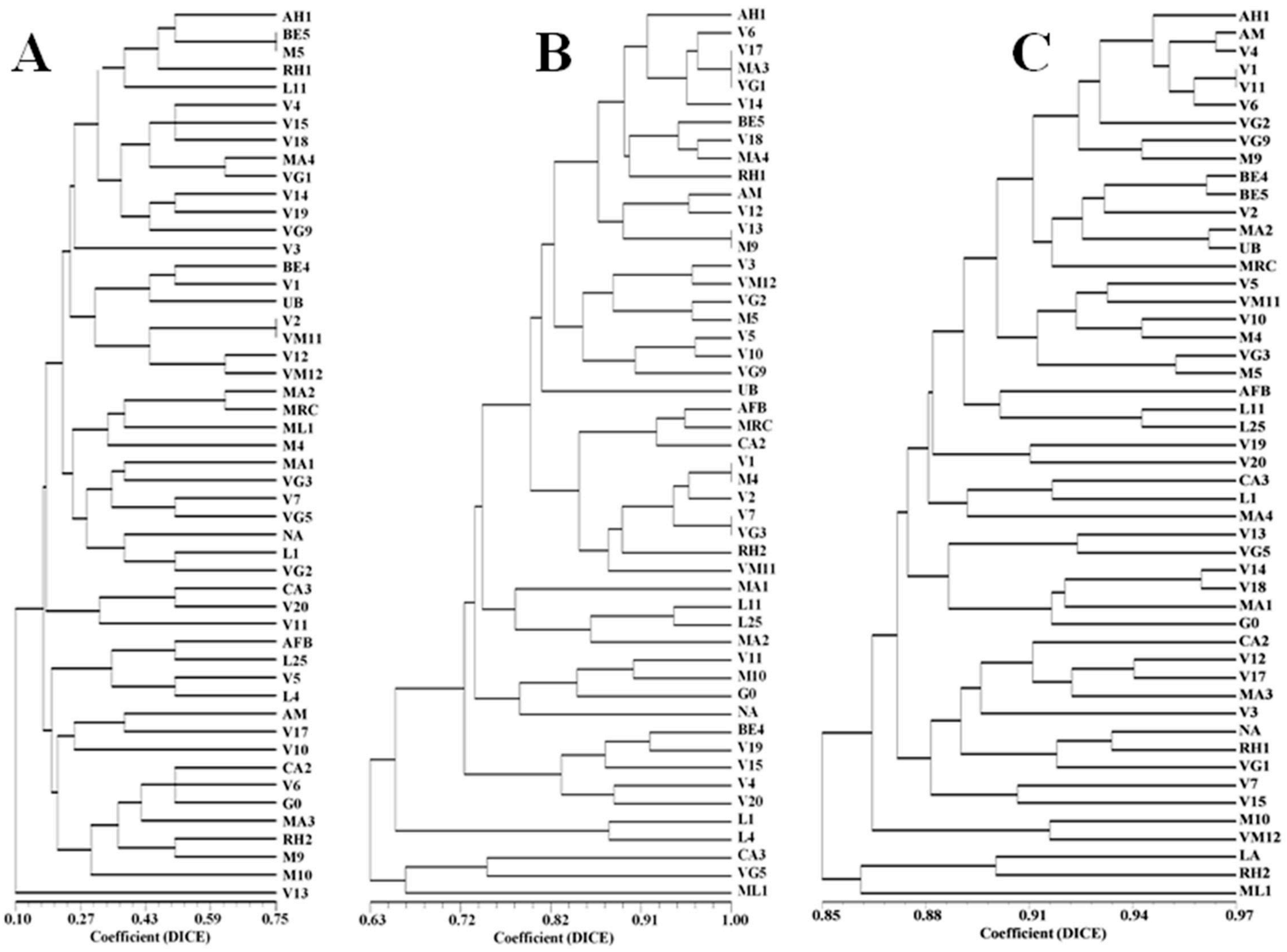


**Supplementary Figure 1**. **(A)** Genetic intraspecific relationships among 50 selected strawberry tree (*Arbutus unedo* L.) germplasm accessions, assessed by SSR markers; **(B)** Genetic relationships among the same accessions assessed by SNP-CAPS marker; **(C)** Genetic relationships among the same accessions, assessed by RAPD and ISSR markers. Notice the very low genetic similarity values estimated by SSR markers *vs.* the estimated by the randomly amplified markers genetic similarity values which, according to multiple studies (see text), are expected to be close or above 0,8 (DICE coefficient).
