# Supplementary material for: Next-Generation Sequencing (NGS) Identified Species-Specific SSR and SNP Markers, Allow the Unequivocal Identification of Strawberry Tree (Arbutus unedo L.) Germplasm Accessions and Contribute to Assess Their Genetic Relationships
Source: Plants (Basel). 2023 Mar 31;12(7):1517. doi: 10.3390/plants12071517 (PMC10096993; doi:10.3390/plants12071517)
Supplement: Supplementary file 1 [file plants-12-01517-s001.zip › plants-2275838-Supplementary Table S1.pdf]

Supplementary Table S1. Morphologic traits of 50 accessions selected for further breeding.

Accession	Leaf shape	Flower colour	Fruit shape	Fruit colour	Accession	Leaf shape	Flower colour	Fruit shape	Fruit colour
**AH1**	Broad elliptic	White	Round	Dark red	**L1**	Broad elliptic	White	Oblate	Dark red
**AFB**	Ovate	White	Oblate	Dark red	**L4**	Narrow elliptic	White	Round	Dark red
**AM**	Broad elliptic	White	Elliptic	Yellow	**L11**	Broad elliptic	White	Round	Dark red
**BE4**	Narrow elliptic	Light rose	Elliptic	Dark red	**L25**	Broad elliptic	White	Oblate	Dark red
**BE5**	Narrow elliptic	White	Round	Dark red	**MA1**	Broad elliptic	White	Round	Dark red
**CA2**	Broad elliptic	White	Oblate	Dark red	**MA2**	Narrow elliptic	Light rose	Round	Dark red
**CA3**	Broad elliptic	White	Round	Dark red	**MA3**	Obovate	White	Round	Dark red
**V1**	Narrow elliptic	White	Round	Dark red	**MA4**	Narrow elliptic	White	Round	Dark red
**V2**	Broad elliptic	White	Oblate	Dark red	**MRC**	Broad elliptic	White	Round	Dark red
**V3**	Narrow elliptic	White	Round	Dark red	**ML1**	Broad elliptic	White	Round	Dark red
**V4**	Narrow elliptic	White	Round	Dark red	**NA**	Broad elliptic	White	Oblate	Dark red
**V5**	Broad elliptic	White	Round	Dark red	**RH1**	Broad elliptic	White	Round	Dark red
**V6**	Narrow elliptic	White	Round	Dark red	**RH2**	Narrow elliptic	White	Round	Dark red
**V7**	Obovate	Light rose	Round	Dark red	**UB**	Broad elliptic	White	Round	Dark red
**V10**	Broad elliptic	White	Oblate	Dark red	**VG1**	Broad elliptic	White	Oblate	Dark red
**V11**	Narrow elliptic	Light rose	Oblate	Dark red	**VG2**	Narrow elliptic	White	Round	Dark red
**V12**	Broad elliptic	Light rose	Oblate	Dark red	**VG3**	Broad elliptic	Light rose	Round	Dark red
**V13**	Narrow elliptic	White	Round	Dark red	**VG5**	Broad elliptic	White	Round	Dark red
**V14**	Broad elliptic	Light rose	Oblate	Orange	**VG9**	Narrow elliptic	White	Round	Dark red
**V15**	Broad elliptic	White	Round	Dark red	**M4**	Broad elliptic	White	Oblate	Dark red
**V17**	Broad elliptic	Light rose	Round	Dark red	**M5**	Broad elliptic	White	Oblate	Dark red
**V18**	Broad elliptic	Light rose	Elliptic	Dark red	**M9**	Broad elliptic	Light rose	Round	Dark red
**V19**	Broad elliptic	White	Round	Dark red	**M10**	Narrow elliptic	Light rose	Round	Dark red
**V20**	Broad elliptic	Light rose	Round	Dark red	**VM11**	Broad elliptic	Light rose	Oblate	Dark red
**G0**	Broad elliptic	Light rose	Round	Dark red	**VM12**	Broad elliptic	White	Round	Dark red
