# Supplementary material for: Next-Generation Sequencing (NGS) Identified Species-Specific SSR and SNP Markers, Allow the Unequivocal Identification of Strawberry Tree (Arbutus unedo L.) Germplasm Accessions and Contribute to Assess Their Genetic Relationships
Source: Plants (Basel). 2023 Mar 31;12(7):1517. doi: 10.3390/plants12071517 (PMC10096993; doi:10.3390/plants12071517)
Supplement: Supplementary file 1 [file plants-12-01517-s001.zip › plants-2275838-Supplementary Table S2.pdf]

Supplementary Table S2. Similarity matrix (SSR markers)

Type: Symmetric (similarity)																																																		
With	50	rows	and	50	columns																																												
	AH1	AFB	AM	BE4	BE5	CA2	CA3	V1	V2	V3	V4	V5	V6	V7	V10	V11	V12	V13	V14	V15	V17	V18	V19	V20	G0	L1	L4	L11	L25	MA1	MA2	MA3	MA4	MRC	ML1	NA	RH1	RH2	UB	VG1	VG2	VG3	VG5	VG9	M4	M5	M9	M10	VM11	VM12
AH1	1.000																																																
AFB	0.250	1.000																																															
AM	0.250	0.000	1.000																																														
BE4	0.250	0.000	0.250	1.000																																													
BE5	0.500	0.125	0.125	0.375	1.000																																												
CA2	0.000	0.000	0.375	0.000	0.125	1.000																																											
CA3	0.000	0.125	0.000	0.125	0.125	0.125	1.000																																										
V1	0.250	0.125	0.125	0.500	0.500	0.125	0.125	1.000																																									
V2	0.250	0.000	0.125	0.250	0.250	0.250	0.000	0.500	1.000																																								
V3	0.125	0.125	0.000	0.250	0.375	0.000	0.250	0.125	0.250	1.000																																							
V4	0.125	0.250	0.000	0.125	0.250	0.250	0.375	0.375	0.250	0.250	1.000																																						
V5	0.375	0.250	0.125	0.375	0.250	0.250	0.125	0.250	0.250	0.250	0.250	1.000																																					
V6	0.125	0.125	0.125	0.250	0.375	0.500	0.250	0.500	0.375	0.250	0.500	0.500	1.000																																				
V7	0.125	0.000	0.125	0.250	0.125	0.125	0.250	0.125	0.250	0.125	0.125	0.125	0.250	1.000																																			
V10	0.125	0.125	0.250	0.000	0.375	0.125	0.250	0.125	0.125	0.250	0.250	0.125	0.125	0.250	1.000																																		
V11	0.250	0.250	0.250	0.375	0.125	0.000	0.375	0.250	0.000	0.000	0.375	0.250	0.125	0.125	0.000	1.000																																	
V12	0.375	0.000	0.125	0.375	0.250	0.000	0.000	0.250	0.375	0.375	0.125	0.250	0.375	0.250	0.125	0.000	1.000																																
V13	0.000	0.000	0.250	0.125	0.000	0.125	0.000	0.000	0.125	0.125	0.000	0.125	0.125	0.250	0.000	0.125	0.000	1.000																															
V14	0.000	0.000	0.250	0.000	0.250	0.500	0.250	0.250	0.375	0.250	0.375	0.000	0.250	0.250	0.250	0.000	0.000	0.125	1.000																														
V15	0.250	0.125	0.000	0.125	0.250	0.125	0.250	0.125	0.250	0.250	0.500	0.250	0.250	0.500	0.125	0.125	0.125	0.125	0.375	1.000																													
V17	0.250	0.125	0.375	0.500	0.250	0.125	0.000	0.250	0.125	0.375	0.125	0.375	0.250	0.125	0.250	0.250	0.250	0.125	0.000	0.000	1.000																												
V18	0.125	0.125	0.000	0.375	0.375	0.000	0.250	0.500	0.375	0.375	0.500	0.250	0.375	0.250	0.125	0.125	0.375	0.125	0.250	0.500	0.125	1.000																											
V19	0.250	0.125	0.375	0.125	0.250	0.250	0.250	0.375	0.500	0.250	0.250	0.250	0.375	0.250	0.125	0.125	0.250	0.250	0.500	0.375	0.000	0.500	1.000																										
V20	0.000	0.000	0.125	0.125	0.125	0.125	0.500	0.000	0.125	0.250	0.250	0.125	0.125	0.375	0.250	0.250	0.000	0.125	0.375	0.500	0.125	0.250	0.250	1.000																									
G0	0.000	0.000	0.250	0.375	0.000	0.500	0.125	0.125	0.250	0.250	0.250	0.375	0.500	0.250	0.000	0.125	0.250	0.250	0.125	0.125	0.500	0.125	0.125	0.125	1.000																								
L1	0.000	0.000	0.000	0.125	0.125	0.250	0.250	0.125	0.250	0.125	0.250	0.125	0.250	0.375	0.125	0.125	0.000	0.125	0.375	0.500	0.000	0.125	0.125	0.500	0.125	1.000																							
L4	0.500	0.375	0.250	0.125	0.250	0.125	0.125	0.250	0.125	0.125	0.250	0.500	0.375	0.125	0.250	0.250	0.125	0.125	0.000	0.250	0.250	0.250	0.375	0.125	0.125	0.000	1.000																						
L11	0.250	0.125	0.000	0.000	0.375	0.375	0.250	0.000	0.125	0.375	0.250	0.250	0.250	0.250	0.250	0.000	0.000	0.125	0.375	0.375	0.250	0.250	0.250	0.500	0.375	0.250	0.125	1.000																					
L25	0.250	0.500	0.250	0.000	0.250	0.250	0.000	0.000	0.000	0.250	0.125	0.375	0.250	0.000	0.125	0.125	0.125	0.125	0.125	0.125	0.125	0.000	0.250	0.000	0.125	0.000	0.375	0.125	1.000																				
MA1	0.125	0.250	0.125	0.250	0.125	0.125	0.250	0.000	0.250	0.250	0.125	0.250	0.125	0.375	0.125	0.250	0.125	0.250	0.250	0.250	0.250	0.250	0.125	0.375	0.375	0.375	0.125	0.375	0.250	1.000																			
MA2	0.375	0.250	0.125	0.000	0.375	0.125	0.125	0.125	0.375	0.375	0.250	0.125	0.125	0.250	0.375	0.000	0.250	0.125	0.375	0.375	0.125	0.250	0.375	0.375	0.000	0.375	0.125	0.375	0.250	0.375	1.000																		
MA3	0.000	0.125	0.250	0.125	0.125	0.375	0.375	0.250	0.250	0.250	0.500	0.250	0.500	0.125	0.250	0.125	0.125	0.125	0.250	0.250	0.250	0.375	0.375	0.375	0.375	0.125	0.375	0.250	0.125	0.125	0.125	1.000																	
MA4	0.250	0.125	0.125	0.250	0.375	0.250	0.375	0.375	0.125	0.125	0.375	0.250	0.500	0.375	0.250	0.250	0.375	0.000	0.250	0.375	0.250	0.500	0.375	0.250	0.250	0.125	0.250	0.375	0.000	0.250	0.125	0.375	1.000																
MRC	0.125	0.125	0.250	0.125	0.250	0.125	0.250	0.250	0.375	0.250	0.375	0.000	0.250	0.375	0.250	0.250	0.125	0.375	0.375	0.375	0.125	0.375	0.500	0.375	0.125	0.375	0.125	0.250	0.000	0.250	0.625	0.250	0.250	1.000															
ML1	0.125	0.375	0.125	0.000	0.000	0.000	0.125	0.125	0.250	0.125	0.125	0.000	0.125	0.125	0.000	0.125	0.125	0.125	0.125	0.125	0.000	0.250	0.375	0.125	0.000	0.125	0.125	0.125	0.125	0.250	0.375	0.125	0.125	0.375	1.000														
NA	0.125	0.000	0.000	0.125	0.250	0.375	0.125	0.250	0.500	0.125	0.250	0.125	0.375	0.375	0.125	0.000	0.125	0.250	0.375	0.375	0.000	0.250	0.250	0.250	0.125	0.375	0.125	0.250	0.000	0.250	0.250	0.250	0.250	0.250	0.250	1.000													
RH1	0.375	0.125	0.125	0.250	0.500	0.000	0.125	0.250	0.250	0.250	0.250	0.250	0.125	0.125	0.250	0.125	0.125	0.000	0.125	0.375	0.250	0.375	0.250	0.250	0.125	0.000	0.250	0.375	0.125	0.250	0.250	0.125	0.375	0.125	0.000	0.125	1.000												
RH2	0.125	0.000	0.375	0.250	0.250	0.375	0.125	0.375	0.250	0.125	0.375	0.125	0.375	0.125	0.250	0.125	0.125	0.000	0.375	0.125	0.375	0.125	0.125	0.250	0.250	0.375	0.125	0.125	0.125	0.125	0.125	0.375	0.250	0.125	0.000	0.250	0.125	1.000											
UB	0.125	0.125	0.250	0.375	0.375	0.250	0.125	0.500	0.250	0.000	0.250	0.250	0.500	0.250	0.125	0.250	0.125	0.250	0.125	0.125	0.125	0.375	0.375	0.000	0.250	0.250	0.375	0.000	0.125	0.125	0.125	0.250	0.250	0.375	0.125	0.250	0.125	0.250	1.000										
VG1	0.375	0.250	0.000	0.125	0.500	0.250	0.375	0.375	0.250	0.250	0.500	0.250	0.500	0.250	0.375	0.125	0.375	0.000	0.375	0.375	0.125	0.500	0.375	0.250	0.125	0.250	0.375	0.375	0.125	0.375	0.500	0.375	0.625	0.375	0.125	0.375	0.375	0.250	0.375	1.000									
VG2	0.125	0.125	0.125	0.250	0.250	0.125	0.250	0.375	0.375	0.125	0.250	0.250	0.375	0.375	0.375	0.250	0.250	0.125	0.250	0.250	0.125	0.375	0.375	0.375	0.000	0.500	0.375	0.125	0.000	0.250	0.250	0.250	0.375	0.375	0.250	0.375	0.125	0.375	0.500	0.375	1.000								
VG3	0.125	0.000	0.375	0.375	0.125	0.375	0.125	0.125	0.125	0.125	0.125	0.125	0.250	0.375	0.000	0.125	0.375	0.125	0.375	0.250	0.250	0.250	0.250	0.125	0.375	0.250	0.125	0.125	0.125	0.375	0.000	0.125	0.375	0.125	0.000	0.250	0.125	0.375	0.375	0.375	0.250	1.000							
VG5	0.375	0.125	0.250	0.250	0.250	0.000	0.000	0.125	0.375	0.250	0.000	0.250	0.125	0.500	0.125	0.125	0.125	0.250	0.250	0.125	0.250	0.250	0.250	0.375	0.250	0.125	0.125	0.250	0.375	0.125	0.375	0.000	0.125	0.375	0.250	0.250	0.375	0.000	0.250	0.250	0.250	0.250	1.000						
VG9	0.250	0.125	0.125	0.250	0.375	0.125	0.125	0.375	0.250	0.125	0.250	0.250	0.125	0.250	0.250	0.250	0.125	0.375	0.000	0.375	0.375	0.000	0.500	0.500	0.125	0.000	0.000	0.250	0.125	0.250	0.125	0.125	0.500	0.125	0.000	0.125	0.250	0.125	0.250	0.375	0.250	0.375	0.125	1.000					
M4	0.125	0.125	0.250	0.000	0.000	0.000	0.125	0.125	0.250	0.125	0.125	0.000	0.125	0.125	0.125	0.125	0.125	0.125	0.125	0.125	0.000	0.250	0.375	0.250	0.000	0.250	0.125	0.250	0.000	0.125	0.250	0.125	0.125	0.375	0.375	0.125	0.125	0.125	0.250	0.125	0.375	0.125	0.500	0.000	1.000				
M5	0.500	0.125	0.125	0.250	0.750	0.250	0.250	0.500	0.375	0.250	0.375	0.250	0.375	0.250	0.375	0.125	0.125	0.000	0.500	0.375	0.250	0.250	0.375	0.250	0.125	0.250	0.250	0.500	0.125	0.250	0.375	0.250	0.500	0.250	0.000	0.375	0.500	0.375	0.250	0.625	0.250	0.250	0.250	0.375	0.000	1.000			
M9	0.125	0.125	0.000	0.250	0.125	0.375	0.250	0.250	0.375	0.125	0.500	0.375	0.375	0.125	0.125	0.250	0.125	0.000	0.250	0.250	0.125	0.125	0.000	0.250	0.500	0.375	0.125	0.250	0.125	0.375	0.125	0.250	0.125	0.125	0.000	0.250	0.125	0.500	0.125	0.250	0.250	0.125	0.000	0.125	0.000	0.250	1.000		
M10	0.125	0.250	0.125	0.125	0.125	0.125	0.125	0.375	0.125	0.250	0.375	0.250	0.375	0.000	0.125	0.250	0.250	0.000	0.000	0.125	0.250	0.250	0.125	0.125	0.250	0.000	0.375	0.125	0.250	0.000	0.000	0.375	0.125	0.125	0.125	0.000	0.000	0.250	0.125	0.125	0.250	0.000	0.000	0.125	0.125	0.000	0.375	1.000	
VM11	0.250	0.000	0.125	0.250	0.250	0.250	0.000	0.375	0.750	0.125	0.250	0.250	0.375	0.125	0.125	0.000	0.375	0.000	0.125	0.125	0.125	0.250	0.250	0.125	0.250	0.125	0.125	0.125	0.000	0.125	0.250	0.250	0.125	0.250	0.125	0.375	0.375	0.250	0.250	0.250	0.250	0.125	0.375	0.125	0.250	0.250	0.375	0.125	1.000	
VM12	0.500	0.000	0.250	0.375	0.250	0.000	0.000	0.375	0.500	0.125	0.125	0.250	0.250	0.125	0.000	0.125	0.625	0.000	0.000	0.125	0.250	0.375	0.250	0.000	0.250	0.125	0.125	0.000	0.000	0.250	0.250	0.125	0.375	0.125	0.125	0.125	0.375	0.375	0.250	0.375	0.250	0.375	0.250	0.250	0.250	0.250	0.250	0.125	0.500	1.000
