# Supplementary material for: Next-Generation Sequencing (NGS) Identified Species-Specific SSR and SNP Markers, Allow the Unequivocal Identification of Strawberry Tree (Arbutus unedo L.) Germplasm Accessions and Contribute to Assess Their Genetic Relationships
Source: Plants (Basel). 2023 Mar 31;12(7):1517. doi: 10.3390/plants12071517 (PMC10096993; doi:10.3390/plants12071517)
Supplement: Supplementary file 1 [file plants-12-01517-s001.zip › plants-2275838-Supplementary Table S3.pdf]

Supplementary Table S3. Similarity matrix (SNP-CAPS)

Type: Symmetric (similarity)
With 50 rows and 50 columns

	AH1	AFB	AM	BE4	BE5	CA2	CA3	V1	V2	V3	V4	V5	V6	V7	V10	V11	V12
AH1	1.000																
AFB	0.750	1.000															
AM	0.800	0.857	1.000														
BE4	0.667	0.609	0.750	1.000													
BE5	0.815	0.870	0.833	0.769	1.000												
CA2	0.696	0.947	0.800	0.636	0.818	1.000											
CA3	0.636	0.444	0.632	0.667	0.571	0.471	1.000										
V1	0.692	0.818	0.783	0.800	0.800	0.857	0.600	1.000									
V2	0.640	0.762	0.727	0.750	0.750	0.800	0.526	0.957	1.000								
V3	0.741	0.696	0.750	0.846	0.846	0.727	0.667	0.880	0.917	1.000							
V4	0.615	0.636	0.609	0.880	0.800	0.667	0.500	0.750	0.696	0.800	1.000						
V5	0.857	0.750	0.800	0.815	0.815	0.783	0.727	0.846	0.800	0.889	0.769	1.000					
V6	0.897	0.800	0.846	0.786	0.929	0.750	0.609	0.815	0.769	0.857	0.741	0.897	1.000				
V7	0.720	0.857	0.818	0.750	0.750	0.900	0.632	0.957	0.909	0.833	0.696	0.880	0.769	1.000			
V10	0.815	0.783	0.833	0.846	0.846	0.818	0.762	0.880	0.833	0.923	0.800	0.963	0.857	0.917	1.000		
V11	0.750	0.700	0.762	0.696	0.696	0.737	0.667	0.727	0.667	0.696	0.636	0.833	0.720	0.762	0.783	1.000	
V12	0.846	0.909	0.957	0.720	0.880	0.857	0.600	0.833	0.783	0.800	0.667	0.846	0.889	0.870	0.880	0.727	1.000
V13	0.880	0.762	0.909	0.750	0.833	0.700	0.737	0.696	0.636	0.750	0.609	0.800	0.846	0.727	0.833	0.762	0.870
V14	0.889	0.783	0.833	0.769	0.846	0.818	0.667	0.800	0.750	0.846	0.720	0.963	0.929	0.833	0.923	0.783	0.880
V15	0.640	0.762	0.727	0.833	0.667	0.800	0.526	0.870	0.818	0.750	0.783	0.800	0.692	0.909	0.833	0.667	0.783
V17	0.929	0.833	0.880	0.741	0.889	0.783	0.636	0.769	0.720	0.815	0.692	0.929	0.966	0.800	0.889	0.750	0.923
V18	0.828	0.800	0.846	0.857	0.929	0.750	0.696	0.889	0.846	0.929	0.815	0.897	0.933	0.846	0.929	0.720	0.889
V19	0.640	0.667	0.818	0.917	0.667	0.700	0.632	0.783	0.727	0.750	0.783	0.800	0.692	0.818	0.833	0.762	0.783
V20	0.667	0.696	0.667	0.846	0.769	0.727	0.476	0.800	0.750	0.769	0.880	0.815	0.786	0.750	0.769	0.783	0.720
G0	0.609	0.632	0.700	0.636	0.636	0.667	0.588	0.667	0.700	0.727	0.571	0.783	0.667	0.700	0.727	0.842	0.667
L1	0.545	0.556	0.737	0.667	0.571	0.588	0.625	0.700	0.737	0.762	0.500	0.727	0.609	0.737	0.762	0.667	0.700
L4	0.455	0.556	0.632	0.667	0.571	0.588	0.500	0.800	0.842	0.762	0.500	0.636	0.609	0.737	0.667	0.556	0.600
L11	0.636	0.778	0.737	0.571	0.762	0.824	0.375	0.700	0.737	0.762	0.600	0.727	0.696	0.737	0.762	0.667	0.800
L25	0.696	0.737	0.800	0.636	0.727	0.778	0.471	0.762	0.800	0.818	0.571	0.783	0.750	0.800	0.818	0.632	0.857
MA1	0.720	0.762	0.727	0.583	0.750	0.700	0.316	0.783	0.818	0.750	0.522	0.720	0.846	0.727	0.667	0.571	0.783
MA2	0.609	0.737	0.700	0.636	0.636	0.778	0.471	0.857	0.900	0.818	0.571	0.783	0.667	0.900	0.818	0.632	0.762
MA3	0.929	0.833	0.880	0.741	0.889	0.783	0.636	0.769	0.720	0.815	0.692	0.929	0.966	0.800	0.889	0.750	0.923
MA4	0.857	0.833	0.880	0.815	0.963	0.783	0.636	0.846	0.800	0.889	0.769	0.857	0.966	0.800	0.889	0.667	0.923
MRC	0.800	0.952	0.909	0.667	0.833	0.900	0.526	0.870	0.818	0.750	0.609	0.800	0.846	0.909	0.833	0.667	0.957
ML1	0.476	0.706	0.667	0.600	0.600	0.750	0.667	0.737	0.667	0.600	0.526	0.667	0.545	0.778	0.700	0.706	0.632
NA	0.783	0.842	0.800	0.636	0.818	0.889	0.588	0.762	0.700	0.727	0.667	0.783	0.750	0.800	0.818	0.842	0.857
RH1	0.828	0.800	0.846	0.786	0.857	0.750	0.609	0.889	0.846	0.857	0.741	0.897	0.933	0.846	0.857	0.800	0.889
RH2	0.769	0.818	0.783	0.720	0.800	0.857	0.500	0.917	0.870	0.800	0.667	0.846	0.889	0.870	0.800	0.727	0.833
UB	0.720	0.667	0.818	0.833	0.833	0.700	0.737	0.783	0.727	0.833	0.696	0.800	0.846	0.727	0.833	0.667	0.783
VG1	0.929	0.833	0.880	0.741	0.889	0.783	0.636	0.769	0.720	0.815	0.692	0.929	0.966	0.800	0.889	0.750	0.923
VG2	0.741	0.783	0.750	0.846	0.923	0.818	0.667	0.880	0.833	0.923	0.880	0.889	0.857	0.833	0.923	0.783	0.800
VG3	0.720	0.857	0.818	0.750	0.750	0.900	0.632	0.957	0.909	0.833	0.696	0.880	0.769	1.000	0.917	0.762	0.870
VG5	0.636	0.667	0.632	0.571	0.571	0.706	0.750	0.800	0.737	0.667	0.500	0.727	0.609	0.842	0.762	0.667	0.700
VG9	0.769	0.636	0.696	0.720	0.720	0.667	0.800	0.750	0.696	0.800	0.667	0.923	0.815	0.783	0.880	0.727	0.750
M4	0.692	0.818	0.783	0.800	0.800	0.857	0.600	1.000	0.957	0.880	0.750	0.846	0.815	0.957	0.880	0.727	0.833
M5	0.692	0.818	0.783	0.800	0.880	0.857	0.600	0.917	0.870	0.880	0.833	0.846	0.815	0.870	0.880	0.818	0.833
M9	0.880	0.762	0.909	0.750	0.833	0.700	0.737	0.696	0.636	0.750	0.609	0.800	0.846	0.727	0.833	0.762	0.870
M10	0.750	0.800	0.857	0.696	0.783	0.842	0.556	0.727	0.667	0.696	0.636	0.833	0.800	0.762	0.783	0.900	0.818
VM11	0.720	0.762	0.818	0.750	0.667	0.800	0.632	0.870	0.818	0.750	0.609	0.880	0.769	0.909	0.833	0.857	0.783
VM12	0.692	0.636	0.696	0.800	0.800	0.667	0.600	0.833	0.870	0.960	0.750	0.846	0.815	0.783	0.880	0.636	0.750

	V13	V14	V15	V17	V18	V19	V20	G0	L1	L4	L11	L25	MA1	MA2	MA3
AH1															
AFB															
AM															
BE4															
BE5															
CA2															
CA3															
V1															
V2															
V3															
V4															
V5															
V6															
V7															
V10															
V11															
V12															
V13	1.000														
V14	0.833	1.000													
V15	0.636	0.750	1.000												
V17	0.880	0.963	0.720	1.000											
V18	0.846	0.857	0.769	0.897	1.000										
V19	0.727	0.750	0.909	0.720	0.769	1.000									
V20	0.583	0.769	0.833	0.741	0.786	0.833	1.000								
G0	0.600	0.727	0.600	0.696	0.667	0.700	0.727	1.000							
L1	0.632	0.667	0.632	0.636	0.696	0.737	0.571	0.706	1.000						
L4	0.526	0.571	0.632	0.545	0.696	0.632	0.571	0.588	0.875	1.000					
L11	0.632	0.762	0.632	0.727	0.696	0.632	0.667	0.706	0.750	0.625	1.000				
L25	0.700	0.818	0.700	0.783	0.750	0.700	0.636	0.667	0.824	0.706	0.941	1.000			
MA1	0.636	0.750	0.636	0.800	0.769	0.545	0.667	0.600	0.632	0.737	0.737	0.800	1.000		
MA2	0.600	0.727	0.800	0.696	0.750	0.700	0.636	0.667	0.824	0.824	0.824	0.889	0.800	1.000	
MA3	0.880	0.963	0.720	1.000	0.897	0.720	0.741	0.696	0.636	0.545	0.727	0.783	0.800	0.696	1.000
MA4	0.880	0.889	0.720	0.929	0.966	0.720	0.741	0.609	0.636	0.636	0.727	0.783	0.800	0.696	0.929
MRC	0.818	0.833	0.818	0.880	0.846	0.727	0.667	0.600	0.632	0.632	0.737	0.800	0.818	0.800	0.880
ML1	0.556	0.600	0.667	0.571	0.636	0.667	0.600	0.750	0.667	0.667	0.533	0.500	0.444	0.625	0.571
NA	0.800	0.818	0.700	0.783	0.750	0.700	0.727	0.667	0.588	0.471	0.824	0.778	0.600	0.667	0.783
RH1	0.769	0.857	0.769	0.897	0.933	0.769	0.857	0.750	0.696	0.696	0.696	0.750	0.846	0.750	0.897
RH2	0.696	0.880	0.783	0.846	0.815	0.696	0.800	0.667	0.600	0.700	0.700	0.762	0.870	0.762	0.846
UB	0.818	0.833	0.636	0.800	0.846	0.727	0.667	0.600	0.737	0.737	0.632	0.700	0.636	0.600	0.800
VG1	0.880	0.963	0.720	1.000	0.897	0.720	0.741	0.696	0.636	0.545	0.727	0.783	0.800	0.696	1.000
VG2	0.750	0.846	0.750	0.815	0.929	0.750	0.846	0.727	0.667	0.667	0.762	0.727	0.667	0.727	0.815
VG3	0.727	0.833	0.909	0.800	0.846	0.818	0.750	0.700	0.737	0.737	0.737	0.800	0.727	0.900	0.800
VG5	0.632	0.667	0.737	0.636	0.696	0.632	0.571	0.588	0.500	0.500	0.500	0.588	0.526	0.706	0.636
VG9	0.696	0.880	0.696	0.846	0.815	0.696	0.720	0.762	0.600	0.500	0.600	0.667	0.609	0.667	0.846
M4	0.696	0.800	0.870	0.769	0.889	0.783	0.800	0.667	0.700	0.800	0.700	0.762	0.783	0.857	0.769
M5	0.696	0.800	0.783	0.769	0.889	0.783	0.880	0.762	0.700	0.700	0.800	0.762	0.696	0.762	0.769
M9	1.000	0.833	0.636	0.880	0.846	0.727	0.583	0.600	0.632	0.526	0.632	0.700	0.636	0.600	0.880
M10	0.762	0.870	0.667	0.833	0.720	0.762	0.783	0.842	0.667	0.556	0.778	0.737	0.667	0.632	0.833
VM11	0.727	0.833	0.818	0.800	0.769	0.818	0.750	0.800	0.737	0.737	0.632	0.700	0.727	0.800	0.800
VM12	0.696	0.800	0.696	0.769	0.889	0.696	0.720	0.667	0.800	0.800	0.800	0.857	0.783	0.857	0.769

	MA4	MRC	ML1	NA	RH1	RH2	UB	VG1	VG2	VG3	VG5	VG9	M4	M5	M9	M10	VM11	VM12
AH1																		
AFB																		
AM																		
BE4																		
BE5																		
CA2																		
CA3																		
V1																		
V2																		
V3																		
V4																		
V5																		
V6																		
V7																		
V10																		
V11																		
V12																		
V13																		
V14																		
V15																		
V17																		
V18																		
V19																		
V20																		
G0																		
L1																		
L4																		
L11																		
L25																		
MA1																		
MA2																		
MA3																		
MA4	1.000																	
MRC	0.880	1.000																
ML1	0.571	0.667	1.000															
NA	0.783	0.800	0.625	1.000														
RH1	0.897	0.846	0.636	0.750	1.000													
RH2	0.846	0.870	0.632	0.762	0.889	1.000												
UB	0.880	0.727	0.667	0.700	0.769	0.783	1.000											
VG1	0.929	0.880	0.571	0.783	0.897	0.846	0.800	1.000										
VG2	0.889	0.750	0.700	0.818	0.857	0.800	0.833	0.815	1.000									
VG3	0.800	0.909	0.778	0.800	0.846	0.870	0.727	0.800	0.833	1.000								
VG5	0.636	0.737	0.667	0.706	0.696	0.700	0.526	0.636	0.667	0.842	1.000							
VG9	0.769	0.696	0.632	0.667	0.815	0.750	0.696	0.846	0.800	0.783	0.800	1.000						
M4	0.846	0.870	0.737	0.762	0.889	0.917	0.783	0.769	0.880	0.957	0.800	0.750	1.000					
M5	0.846	0.783	0.737	0.857	0.889	0.833	0.783	0.769	0.960	0.870	0.700	0.750	0.917	1.000				
M9	0.880	0.818	0.556	0.800	0.769	0.696	0.818	0.880	0.750	0.727	0.632	0.696	0.696	0.696	1.000			
M10	0.750	0.762	0.706	0.842	0.800	0.818	0.762	0.833	0.783	0.762	0.556	0.727	0.727	0.818	0.762	1.000		
VM11	0.720	0.818	0.778	0.700	0.846	0.870	0.727	0.800	0.750	0.909	0.737	0.783	0.870	0.783	0.727	0.857	1.000	
VM12	0.846	0.696	0.526	0.667	0.815	0.750	0.783	0.769	0.880	0.783	0.600	0.750	0.833	0.833	0.696	0.636	0.696	1.000
