# Supplementary material for: Next-Generation Sequencing (NGS) Identified Species-Specific SSR and SNP Markers, Allow the Unequivocal Identification of Strawberry Tree (Arbutus unedo L.) Germplasm Accessions and Contribute to Assess Their Genetic Relationships
Source: Plants (Basel). 2023 Mar 31;12(7):1517. doi: 10.3390/plants12071517 (PMC10096993; doi:10.3390/plants12071517)
Supplement: Supplementary file 1 [file plants-12-01517-s001.zip › plants-2275838-Supplementary Table S4.pdf]

Supplementary Table 4. Similarity matrix (SSR and SNP-CAPS)

Type: Symmetric (similarity)
With 50 rows and 50 columns

	AH1	AFB	AM	BE4	BE5	CA2	CA3	V1	V2	V3	V4	V5	V6	V7	V10	V11	V12	V13	V14	V15	V17	V18	V19	V20	G0	L1	L4	L11	L25	MA1	MA2	MA3	MA4	MRC	ML1	NA	RH1	RH2	UB	VG1	VG2	VG3	VG5	VG9	M4	M5	M9	M10	VM11	VM12
AH1	1.000																																																	
AFB	0.550	1.000																																																
AM	0.585	0.486	1.000																																															
BE4	0.512	0.359	0.550	1.000																																														
BE5	0.698	0.564	0.550	0.619	1.000																																													
CA2	0.410	0.514	0.611	0.368	0.526	1.000																																												
CA3	0.368	0.294	0.343	0.432	0.378	0.303	1.000																																											
V1	0.524	0.526	0.513	0.683	0.683	0.541	0.389	1.000																																										
V2	0.488	0.432	0.474	0.550	0.550	0.556	0.286	0.769	1.000																																									
V3	0.512	0.462	0.450	0.619	0.667	0.421	0.486	0.585	0.650	1.000																																								
V4	0.429	0.474	0.359	0.585	0.585	0.486	0.444	0.600	0.513	0.585	1.000																																							
V5	0.682	0.550	0.537	0.651	0.605	0.564	0.474	0.619	0.585	0.651	0.571	1.000																																						
V6	0.622	0.537	0.571	0.591	0.727	0.650	0.462	0.698	0.619	0.636	0.651	0.756	1.000																																					
V7	0.488	0.486	0.526	0.550	0.500	0.556	0.457	0.615	0.632	0.550	0.462	0.585	0.571	1.000																																				
V10	0.558	0.513	0.600	0.524	0.667	0.526	0.541	0.585	0.550	0.667	0.585	0.651	0.591	0.650	1.000																																			
V11	0.550	0.500	0.541	0.564	0.462	0.400	0.529	0.526	0.378	0.410	0.526	0.600	0.488	0.486	0.462	1.000																																		
V12	0.667	0.526	0.615	0.585	0.634	0.486	0.333	0.600	0.615	0.634	0.450	0.619	0.698	0.615	0.585	0.421	1.000																																	
V13	0.537	0.432	0.632	0.500	0.500	0.444	0.400	0.410	0.421	0.500	0.359	0.537	0.571	0.526	0.500	0.486	0.513	1.000																																
V14	0.558	0.462	0.600	0.476	0.619	0.684	0.486	0.585	0.600	0.619	0.585	0.605	0.682	0.600	0.667	0.462	0.537	0.550	1.000																															
V15	0.488	0.486	0.421	0.550	0.500	0.500	0.400	0.564	0.579	0.550	0.667	0.585	0.524	0.737	0.550	0.432	0.513	0.421	0.600	1.000																														
V17	0.682	0.550	0.683	0.651	0.651	0.513	0.368	0.571	0.488	0.651	0.476	0.727	0.711	0.537	0.651	0.550	0.667	0.585	0.605	0.439	1.000																													
V18	0.578	0.537	0.524	0.682	0.727	0.450	0.513	0.744	0.667	0.727	0.698	0.667	0.739	0.619	0.636	0.488	0.698	0.571	0.636	0.667	0.622	1.000																												
V19	0.488	0.432	0.632	0.600	0.500	0.500	0.457	0.615	0.632	0.550	0.564	0.585	0.571	0.579	0.550	0.486	0.564	0.526	0.650	0.684	0.439	0.667	1.000																											
V20	0.419	0.410	0.450	0.571	0.524	0.474	0.486	0.488	0.500	0.571	0.634	0.558	0.545	0.600	0.571	0.564	0.439	0.400	0.619	0.700	0.512	0.591	0.600	1.000																										
G0	0.359	0.343	0.500	0.526	0.368	0.588	0.364	0.432	0.500	0.526	0.432	0.615	0.600	0.500	0.421	0.514	0.486	0.444	0.474	0.389	0.615	0.450	0.444	0.474	1.000																									
L1	0.316	0.294	0.400	0.432	0.378	0.424	0.438	0.444	0.514	0.486	0.389	0.474	0.462	0.571	0.486	0.412	0.389	0.400	0.541	0.571	0.368	0.462	0.457	0.541	0.424	1.000																								
L4	0.474	0.471	0.457	0.432	0.432	0.364	0.313	0.556	0.514	0.486	0.389	0.579	0.513	0.457	0.486	0.412	0.389	0.343	0.324	0.457	0.421	0.513	0.514	0.378	0.364	0.438	1.000																							
L11	0.474	0.471	0.400	0.324	0.595	0.606	0.313	0.389	0.457	0.595	0.444	0.526	0.513	0.514	0.541	0.353	0.444	0.400	0.595	0.514	0.526	0.513	0.457	0.595	0.545	0.500	0.375	1.000																						
L25	0.513	0.629	0.556	0.368	0.526	0.529	0.242	0.432	0.444	0.579	0.378	0.615	0.550	0.444	0.526	0.400	0.541	0.444	0.526	0.444	0.513	0.450	0.500	0.368	0.412	0.424	0.545	0.545	1.000																					
MA1	0.488	0.541	0.474	0.450	0.500	0.444	0.286	0.462	0.579	0.550	0.359	0.537	0.571	0.579	0.450	0.432	0.513	0.474	0.550	0.474	0.585	0.571	0.368	0.550	0.500	0.514	0.457	0.571	0.556	1.000																				
MA2	0.513	0.514	0.444	0.368	0.526	0.471	0.303	0.541	0.667	0.632	0.432	0.513	0.450	0.611	0.632	0.343	0.541	0.389	0.579	0.611	0.462	0.550	0.556	0.526	0.353	0.606	0.485	0.606	0.588	0.611	1.000																			
MA3	0.591	0.550	0.634	0.512	0.605	0.615	0.526	0.571	0.537	0.605	0.619	0.682	0.800	0.537	0.651	0.500	0.619	0.585	0.698	0.537	0.727	0.711	0.585	0.605	0.564	0.421	0.474	0.526	0.513	0.537	0.462	1.000																		
MA4	0.636	0.550	0.585	0.605	0.744	0.564	0.526	0.667	0.537	0.605	0.619	0.636	0.800	0.634	0.651	0.500	0.714	0.537	0.651	0.585	0.682	0.800	0.585	0.558	0.462	0.421	0.474	0.579	0.462	0.585	0.462	0.727	1.000																	
MRC	0.537	0.595	0.632	0.450	0.600	0.556	0.400	0.615	0.632	0.550	0.513	0.488	0.619	0.684	0.600	0.486	0.615	0.632	0.650	0.632	0.585	0.667	0.632	0.550	0.389	0.514	0.400	0.514	0.444	0.579	0.722	0.634	0.634	1.000																
ML1	0.324	0.545	0.412	0.333	0.333	0.375	0.387	0.457	0.471	0.389	0.343	0.378	0.368	0.471	0.389	0.424	0.400	0.353	0.389	0.412	0.324	0.474	0.529	0.389	0.375	0.387	0.387	0.323	0.313	0.353	0.500	0.378	0.378	0.529	1.000															
NA	0.513	0.457	0.444	0.421	0.579	0.647	0.364	0.541	0.611	0.474	0.486	0.513	0.600	0.611	0.526	0.457	0.541	0.556	0.632	0.556	0.462	0.550	0.500	0.526	0.412	0.485	0.303	0.545	0.412	0.444	0.471	0.564	0.564	0.556	0.438	1.000														
RH1	0.667	0.537	0.571	0.591	0.727	0.450	0.410	0.651	0.619	0.636	0.558	0.667	0.652	0.571	0.636	0.537	0.605	0.476	0.591	0.619	0.667	0.739	0.571	0.636	0.500	0.410	0.513	0.564	0.500	0.619	0.550	0.622	0.711	0.571	0.368	0.500	1.000													
RH2	0.524	0.474	0.615	0.537	0.585	0.649	0.333	0.700	0.615	0.537	0.550	0.571	0.698	0.564	0.585	0.474	0.550	0.410	0.683	0.513	0.667	0.558	0.462	0.585	0.486	0.500	0.444	0.444	0.486	0.564	0.486	0.667	0.619	0.564	0.343	0.541	0.605	1.000												
UB	0.488	0.432	0.579	0.650	0.650	0.500	0.457	0.667	0.526	0.500	0.513	0.585	0.714	0.526	0.550	0.486	0.513	0.579	0.550	0.421	0.537	0.667	0.579	0.400	0.444	0.514	0.571	0.343	0.444	0.421	0.389	0.585	0.634	0.579	0.412	0.500	0.524	0.564	1.000											
VG1	0.727	0.600	0.537	0.512	0.744	0.564	0.526	0.619	0.537	0.605	0.619	0.682	0.800	0.585	0.698	0.500	0.714	0.537	0.744	0.585	0.682	0.756	0.585	0.558	0.462	0.474	0.474	0.579	0.513	0.634	0.615	0.773	0.818	0.683	0.378	0.615	0.711	0.619	0.634	1.000										
VG2	0.512	0.513	0.500	0.619	0.667	0.526	0.486	0.683	0.650	0.619	0.634	0.651	0.682	0.650	0.714	0.564	0.585	0.500	0.619	0.550	0.558	0.727	0.600	0.667	0.421	0.595	0.541	0.486	0.421	0.500	0.526	0.605	0.698	0.600	0.500	0.632	0.591	0.634	0.700	0.651	1.000									
VG3	0.488	0.486	0.632	0.600	0.500	0.667	0.400	0.615	0.579	0.550	0.462	0.585	0.571	0.737	0.550	0.486	0.667	0.474	0.650	0.632	0.585	0.619	0.579	0.500	0.556	0.514	0.457	0.457	0.500	0.579	0.500	0.537	0.634	0.579	0.412	0.556	0.571	0.667	0.579	0.634	0.600	1.000								
VG5	0.526	0.412	0.457	0.432	0.432	0.364	0.375	0.500	0.571	0.486	0.278	0.526	0.410	0.686	0.486	0.412	0.500	0.457	0.432	0.514	0.474	0.513	0.514	0.432	0.364	0.313	0.375	0.438	0.364	0.457	0.545	0.368	0.421	0.571	0.452	0.485	0.564	0.389	0.400	0.474	0.486	0.571	1.000							
VG9	0.571	0.421	0.462	0.537	0.585	0.432	0.500	0.600	0.513	0.537	0.500	0.667	0.558	0.564	0.634	0.474	0.600	0.410	0.683	0.564	0.524	0.698	0.615	0.488	0.432	0.333	0.389	0.389	0.486	0.410	0.432	0.571	0.667	0.462	0.343	0.432	0.605	0.500	0.513	0.667	0.585	0.615	0.500	1.000						
M4	0.476	0.526	0.564	0.488	0.488	0.486	0.389	0.650	0.667	0.585	0.500	0.524	0.558	0.615	0.585	0.474	0.550	0.462	0.537	0.564	0.476	0.651	0.615	0.585	0.378	0.500	0.500	0.500	0.432	0.513	0.595	0.524	0.571	0.667	0.571	0.486	0.605	0.600	0.564	0.524	0.683	0.615	0.667	0.450	1.000					
M5	0.619	0.526	0.513	0.585	0.829	0.595	0.444	0.750	0.667	0.634	0.650	0.619	0.651	0.615	0.683	0.526	0.550	0.410	0.683	0.615	0.571	0.651	0.615	0.634	0.486	0.500	0.500	0.667	0.486	0.513	0.595	0.571	0.714	0.564	0.400	0.649	0.744	0.650	0.564	0.714	0.683	0.615	0.500	0.600	0.550	1.000				
M9	0.585	0.486	0.526	0.550	0.550	0.556	0.514	0.513	0.526	0.500	0.564	0.634	0.667	0.474	0.550	0.541	0.564	0.579	0.600	0.474	0.585	0.571	0.421	0.450	0.556	0.514	0.343	0.457	0.444	0.526	0.389	0.634	0.585	0.526	0.294	0.556	0.524	0.615	0.526	0.634	0.550	0.474	0.343	0.462	0.410	0.513	1.000			
M10	0.500	0.556	0.541	0.462	0.513	0.514	0.353	0.579	0.432	0.513	0.526	0.600	0.634	0.432	0.513	0.611	0.579	0.432	0.513	0.432	0.600	0.537	0.486	0.513	0.571	0.353	0.471	0.471	0.514	0.378	0.343	0.650	0.500	0.486	0.424	0.457	0.488	0.579	0.486	0.550	0.564	0.432	0.294	0.474	0.474	0.474	0.595	1.000		
VM11	0.537	0.432	0.526	0.550	0.500	0.556	0.343	0.667	0.789	0.500	0.462	0.634	0.619	0.579	0.550	0.486	0.615	0.421	0.550	0.526	0.537	0.571	0.579	0.500	0.556	0.457	0.457	0.400	0.389	0.474	0.556	0.585	0.488	0.579	0.471	0.556	0.667	0.615	0.526	0.585	0.550	0.579	0.571	0.513	0.615	0.564	0.579	0.541	1.000	
VM12	0.619	0.368	0.513	0.634	0.585	0.378	0.333	0.650	0.718	0.634	0.500	0.619	0.605	0.513	0.537	0.421	0.700	0.410	0.488	0.462	0.571	0.698	0.513	0.439	0.486	0.500	0.500	0.444	0.486	0.564	0.595	0.524	0.667	0.462	0.343	0.432	0.651	0.600	0.564	0.619	0.634	0.615	0.444	0.550	0.600	0.600	0.513	0.421	0.615	1.000
