# Supplementary material for: Next-Generation Sequencing (NGS) Identified Species-Specific SSR and SNP Markers, Allow the Unequivocal Identification of Strawberry Tree (Arbutus unedo L.) Germplasm Accessions and Contribute to Assess Their Genetic Relationships
Source: Plants (Basel). 2023 Mar 31;12(7):1517. doi: 10.3390/plants12071517 (PMC10096993; doi:10.3390/plants12071517)
Supplement: Supplementary file 1 [file plants-12-01517-s001.zip › plants-2275838-Supplementary Table S6.pdf]

Supplementary Table S5. SSR, SNP-CAPS, RAPD and ISSR markers

Type: Symmetric similarity
With 50 rows and 50 columns

	AH1	AFB	AM	BE4	BE5	CA2	CA3	V1	V2	V3	V4	V5	V6	V7	V10	V11	V12	V13	V14	V15	V17	V18	V19	V20	G0
AH1	1.000																								
AFB	0.823	1.000																							
AM	0.840	0.794	1.000																						
BE4	0.784	0.734	0.838	1.000																					
BE5	0.838	0.791	0.838	0.863	1.000																				
CA2	0.766	0.773	0.809	0.705	0.763	1.000																			
CA3	0.763	0.738	0.769	0.754	0.738	0.732	1.000																		
V1	0.827	0.780	0.840	0.851	0.838	0.780	0.763	1.000																	
V2	0.761	0.756	0.785	0.830	0.806	0.768	0.718	0.851	1.000																
V3	0.696	0.660	0.719	0.754	0.779	0.705	0.722	0.750	0.768	1.000															
V4	0.808	0.803	0.808	0.832	0.832	0.803	0.779	0.848	0.815	0.772	1.000														
V5	0.824	0.777	0.797	0.808	0.795	0.820	0.760	0.797	0.812	0.779	0.819	1.000													
V6	0.850	0.806	0.850	0.821	0.861	0.806	0.791	0.876	0.818	0.783	0.870	0.848	1.000												
V7	0.761	0.752	0.775	0.771	0.743	0.797	0.742	0.817	0.813	0.748	0.783	0.800	0.814	1.000											
V10	0.792	0.771	0.819	0.776	0.830	0.786	0.791	0.792	0.821	0.789	0.827	0.844	0.816	0.794	1.000										
V11	0.835	0.769	0.841	0.812	0.783	0.763	0.800	0.849	0.748	0.725	0.835	0.803	0.831	0.788	0.774	1.000									
V12	0.836	0.774	0.808	0.778	0.792	0.803	0.724	0.808	0.794	0.818	0.789	0.833	0.846	0.826	0.786	0.785	1.000								
V13	0.742	0.699	0.794	0.748	0.748	0.726	0.699	0.742	0.690	0.729	0.712	0.754	0.785	0.768	0.738	0.763	0.766	1.000							
V14	0.792	0.735	0.792	0.755	0.797	0.830	0.742	0.792	0.803	0.757	0.814	0.803	0.816	0.803	0.839	0.742	0.794	0.784	1.000						
V15	0.752	0.727	0.738	0.763	0.748	0.758	0.720	0.794	0.772	0.748	0.803	0.777	0.792	0.857	0.757	0.797	0.774	0.714	0.765	1.000					
V17	0.822	0.759	0.808	0.778	0.778	0.803	0.703	0.781	0.742	0.804	0.776	0.861	0.819	0.797	0.800	0.794	0.859	0.760	0.809	0.759	1.000				
V18	0.805	0.771	0.792	0.830	0.844	0.757	0.760	0.859	0.830	0.814	0.867	0.803	0.855	0.823	0.824	0.774	0.841	0.785	0.861	0.786	0.800	1.000			
V19	0.755	0.746	0.825	0.809	0.780	0.776	0.754	0.797	0.831	0.703	0.806	0.794	0.767	0.785	0.775	0.738	0.763	0.732	0.812	0.761	0.719	0.817	1.000		
V20	0.753	0.745	0.781	0.792	0.792	0.774	0.762	0.767	0.779	0.691	0.816	0.806	0.779	0.768	0.814	0.776	0.718	0.693	0.800	0.774	0.732	0.786	0.820	1.000	
G0	0.752	0.742	0.766	0.748	0.705	0.818	0.727	0.752	0.778	0.705	0.775	0.820	0.806	0.782	0.771	0.775	0.774	0.738	0.800	0.727	0.818	0.771	0.746	0.788	1.000
L1	0.687	0.656	0.733	0.729	0.744	0.748	0.783	0.733	0.759	0.680	0.727	0.760	0.746	0.764	0.769	0.738	0.703	0.724	0.762	0.754	0.677	0.733	0.758	0.772	0.738
L4	0.756	0.778	0.741	0.737	0.737	0.714	0.690	0.756	0.750	0.653	0.735	0.767	0.754	0.724	0.761	0.694	0.702	0.684	0.723	0.683	0.702	0.776	0.750	0.748	0.730
L11	0.803	0.797	0.789	0.714	0.786	0.812	0.760	0.761	0.778	0.729	0.811	0.786	0.800	0.761	0.809	0.744	0.754	0.672	0.779	0.722	0.754	0.766	0.770	0.812	0.797
L25	0.800	0.824	0.828	0.727	0.769	0.824	0.726	0.772	0.775	0.752	0.795	0.839	0.811	0.759	0.819	0.773	0.809	0.720	0.791	0.735	0.780	0.778	0.768	0.766	0.779
MA1	0.769	0.746	0.741	0.738	0.752	0.761	0.661	0.769	0.791	0.722	0.736	0.794	0.767	0.800	0.761	0.758	0.791	0.736	0.826	0.761	0.820	0.803	0.721	0.777	0.806
MA2	0.786	0.794	0.800	0.783	0.811	0.779	0.746	0.828	0.855	0.800	0.808	0.783	0.784	0.818	0.819	0.761	0.809	0.740	0.814	0.779	0.752	0.819	0.812	0.766	0.721
MA3	0.792	0.771	0.805	0.762	0.803	0.814	0.769	0.779	0.776	0.779	0.813	0.844	0.882	0.794	0.824	0.797	0.841	0.779	0.819	0.786	0.855	0.811	0.775	0.786	0.786
MA4	0.797	0.777	0.797	0.795	0.836	0.763	0.797	0.811	0.809	0.765	0.819	0.795	0.874	0.814	0.844	0.765	0.819	0.744	0.825	0.763	0.792	0.884	0.794	0.750	0.734
MRC	0.781	0.774	0.863	0.792	0.819	0.759	0.772	0.836	0.824	0.739	0.803	0.750	0.832	0.797	0.800	0.800	0.789	0.813	0.800	0.759	0.732	0.814	0.820	0.789	0.701
ML1	0.716	0.800	0.746	0.712	0.697	0.752	0.746	0.761	0.723	0.646	0.726	0.742	0.730	0.778	0.722	0.746	0.738	0.723	0.713	0.736	0.723	0.737	0.756	0.723	0.752
NA	0.764	0.741	0.778	0.761	0.803	0.859	0.720	0.806	0.806	0.716	0.786	0.789	0.789	0.824	0.783	0.782	0.786	0.778	0.806	0.785	0.771	0.783	0.818	0.800	0.756
RH1	0.821	0.775	0.821	0.819	0.872	0.775	0.731	0.834	0.809	0.800	0.816	0.832	0.831	0.797	0.840	0.817	0.803	0.756	0.795	0.817	0.830	0.853	0.778	0.816	0.761
RH2	0.757	0.748	0.786	0.768	0.768	0.794	0.683	0.800	0.778	0.692	0.780	0.768	0.811	0.758	0.777	0.719	0.750	0.661	0.785	0.702	0.794	0.763	0.737	0.779	0.748
UB	0.776	0.768	0.844	0.855	0.841	0.768	0.787	0.844	0.812	0.732	0.824	0.800	0.853	0.763	0.795	0.785	0.769	0.797	0.794	0.696	0.741	0.836	0.814	0.741	0.754
VG1	0.830	0.783	0.776	0.772	0.841	0.797	0.760	0.789	0.767	0.779	0.811	0.814	0.840	0.763	0.836	0.774	0.825	0.723	0.817	0.754	0.811	0.822	0.757	0.769	0.739
VG2	0.786	0.763	0.814	0.826	0.826	0.794	0.760	0.857	0.823	0.769	0.851	0.812	0.853	0.803	0.835	0.822	0.809	0.754	0.806	0.763	0.765	0.849	0.782	0.809	0.748
VG3	0.767	0.759	0.836	0.819	0.764	0.818	0.750	0.822	0.824	0.727	0.803	0.806	0.819	0.841	0.814	0.809	0.803	0.744	0.800	0.788	0.775	0.814	0.777	0.775	0.803
VG5	0.780	0.712	0.780	0.777	0.777	0.727	0.752	0.809	0.794	0.712	0.732	0.763	0.764	0.812	0.771	0.782	0.759	0.794	0.785	0.773	0.745	0.800	0.746	0.745	0.742
VG9	0.822	0.759	0.808	0.792	0.806	0.774	0.794	0.808	0.788	0.757	0.830	0.833	0.819	0.783	0.814	0.791	0.831	0.709	0.843	0.759	0.775	0.841	0.791	0.761	0.759
M4	0.781	0.774	0.822	0.778	0.778	0.774	0.772	0.822	0.833	0.745	0.803	0.806	0.819	0.812	0.841	0.785	0.775	0.734	0.800	0.774	0.761	0.828	0.791	0.803	0.745
M5	0.811	0.763	0.824	0.822	0.877	0.820	0.769	0.878	0.842	0.786	0.859	0.836	0.834	0.814	0.844	0.826	0.792	0.733	0.817	0.791	0.792	0.830	0.809	0.819	0.777
M9	0.830	0.768	0.830	0.800	0.814	0.797	0.813	0.803	0.773	0.764	0.838	0.814	0.867	0.777	0.808	0.838	0.811	0.791	0.809	0.754	0.797	0.808	0.729	0.755	0.797
M10	0.766	0.758	0.780	0.734	0.748	0.803	0.689	0.809	0.720	0.686	0.789	0.806	0.792	0.767	0.757	0.815	0.788	0.748	0.770	0.727	0.788	0.757	0.776	0.774	0.773
VM11	0.808	0.745	0.808	0.792	0.764	0.788	0.746	0.849	0.870	0.716	0.789	0.847	0.832	0.797	0.814	0.806	0.817	0.724	0.786	0.774	0.789	0.800	0.777	0.789	0.818
VM12	0.809	0.712	0.766	0.777	0.777	0.758	0.726	0.809	0.816	0.743	0.775	0.806	0.806	0.767	0.786	0.758	0.818	0.704	0.741	0.742	0.759	0.800	0.746	0.745	0.727

	L1	L4	L11	L25	MA1	MA2	MA3	MA4	MRC	ML1	NA	RH1	RH2	UB	VG1	VG2	VG3	VG5	VG9	M4	M5	M9	M10	VM11	VM12
L1	1.000																								
L4	0.701	1.000																							
L11	0.748	0.756	1.000																						
L25	0.724	0.800	0.847	1.000																					
MA1	0.742	0.734	0.756	0.783	1.000																				
MA2	0.787	0.769	0.803	0.814	0.812	1.000																			
MA3	0.723	0.716	0.766	0.778	0.775	0.764	1.000																		
MA4	0.738	0.737	0.786	0.769	0.766	0.797	0.830	1.000																	
MRC	0.766	0.718	0.783	0.766	0.748	0.865	0.786	0.806	1.000																
ML1	0.713	0.723	0.714	0.713	0.709	0.775	0.737	0.697	0.754	1.000															
NA	0.768	0.698	0.779	0.763	0.774	0.806	0.811	0.775	0.786	0.781	1.000														
RH1	0.727	0.765	0.769	0.781	0.819	0.822	0.813	0.832	0.789	0.741	0.814	1.000													
RH2	0.711	0.768	0.773	0.770	0.737	0.756	0.806	0.768	0.735	0.726	0.776	0.766	1.000												
UB	0.760	0.773	0.734	0.775	0.729	0.817	0.781	0.828	0.839	0.733	0.780	0.784	0.759	1.000											
VG1	0.719	0.758	0.806	0.775	0.800	0.817	0.849	0.828	0.783	0.718	0.823	0.865	0.803	0.792	1.000										
VG2	0.754	0.752	0.758	0.770	0.752	0.815	0.791	0.812	0.824	0.758	0.821	0.809	0.769	0.861	0.788	1.000									
VG3	0.756	0.733	0.768	0.809	0.777	0.794	0.772	0.806	0.803	0.754	0.800	0.803	0.824	0.811	0.797	0.824	1.000								
VG5	0.732	0.730	0.722	0.735	0.776	0.824	0.700	0.748	0.803	0.736	0.770	0.817	0.687	0.768	0.739	0.779	0.803	1.000							
VG9	0.688	0.718	0.768	0.809	0.734	0.780	0.786	0.806	0.789	0.692	0.729	0.789	0.735	0.811	0.797	0.809	0.803	0.774	1.000						
M4	0.775	0.748	0.783	0.780	0.763	0.809	0.800	0.819	0.817	0.785	0.771	0.816	0.779	0.811	0.769	0.809	0.817	0.818	0.775	1.000					
M5	0.760	0.737	0.814	0.797	0.780	0.839	0.776	0.822	0.806	0.742	0.845	0.872	0.797	0.828	0.828	0.855	0.861	0.806	0.819	0.806	1.000				
M9	0.766	0.697	0.777	0.775	0.771	0.761	0.836	0.786	0.797	0.702	0.780	0.797	0.774	0.806	0.819	0.788	0.769	0.739	0.811	0.797	0.800	1.000			
M10	0.689	0.730	0.752	0.779	0.746	0.735	0.814	0.719	0.759	0.736	0.800	0.761	0.748	0.754	0.754	0.763	0.730	0.697	0.759	0.759	0.763	0.797	1.000		
VM11	0.740	0.733	0.754	0.780	0.777	0.794	0.814	0.764	0.789	0.769	0.786	0.816	0.809	0.797	0.769	0.794	0.831	0.788	0.789	0.845	0.833	0.825	0.774	1.000	
VM12	0.748	0.746	0.752	0.779	0.761	0.794	0.771	0.806	0.759	0.704	0.741	0.817	0.748	0.768	0.768	0.779	0.774	0.742	0.788	0.803	0.777	0.768	0.773	0.788	1.000
